# Study of the Photoinduced Charge Injection in the Reaction Intermediate of the Dehydrogenation of Formic Acid on Palladium

**DOI:** 10.1002/jcc.70087

**Published:** 2025-03-26

**Authors:** L. Biancorosso, E. Coccia

**Affiliations:** ^1^ Dipartimento di Scienze Chimiche e Farmaceutiche Università di Trieste Trieste Italy

**Keywords:** photocatalysis, real‐time methods, time‐dependent Schroedinger equation

## Abstract

The production rate of hydrogen from formic acid on palladium is enhanced in the presence of an Au nanorod by irradiating the system at its plasmon frequency. Taking inspiration from this, we study here the effect of the shape of the Pd cluster (from Pd(111)) on the photoinduced charge injection into the HCOO moiety and adsorbed H, which are the reaction intermediates of the dehydrogenation of formic acid, upon irradiation with a pulse with a carrier frequency equal to the plasmon resonance of a (not included) Au nanorod. We simulate the electron/hole dynamics at frozen nuclei by propagating the time‐dependent Schrödinger equation in the space of time‐dependent density‐functional‐theory pseudo‐eigenstates in the tight‐binding approximation. We have taken into account a cluster with two layers of Pd and 3×3 and 4×4 atoms per layer (2L3 and 2L4, respectively) or with three layers and 3×3 atoms per layer (3L3). For all the systems, a net negative charge on HCOO has been found, according to a photoinduced direct charge‐transfer mechanism. For 3L3, an indirect charge‐transfer mechanism, occurring after 50 fs and inducing a hole injection into HCOO, has also been found. Moreover, we also used a tailored pulse to populate the antibonding molecular orbital localized on the C‐H bond for 3L3.

## Graphical abstract

A real‐time method based on the propagation of the time‐dependent Schrödinger equation in the space of DFT and TDDFT states within the tight‐binding approximation has been applied to study the electron injection into HCOO from Pd atoms, and the charge distribution among in the Pd cluster.

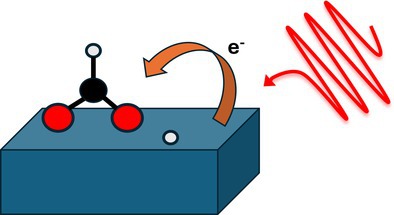

## 1 Introduction

The depletion of fossil fuels and their environmental harm have spurred research into sustainable, eco‐friendly energy alternatives. Solutions like solar cells, biomass conversion, and green fuels are being extensively explored and used [1, 2, 3, 4]. Among these, hydrogen stands out as a clean fuel meeting both sustainability and environmental requirements [5, 6]. However, challenges in its storage and transportation persist.

Formic acid HCOOH (FA) emerges as a promising, safe and abundant hydrogen reservoir [7, 8, 9, 10, 11, 12]. HCOOH decompositon (FAD) becomes a key reaction in catalysis and energy storage, particularly in hydrogen production [13, 14, 15, 16, 17]. This reaction occurs through two main pathways: Dehydrogenation, which produces carbon dioxide (CO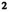) and hydrogen (H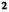), and dehydration, which provides carbon monoxide (CO) and water (H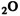) [18, 19, 20, 21]. The two pathways are energetically competitive, and many efforts have been made for improving the selectivity of the reaction towards hydrogen production [22, 23]. The mechanism through which FAD brings to the formation of H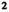 and CO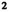 is a crucial step which has been thoroughly investigated by theory [21, 22, 23, 24, 25, 26, 27, 28, 29]. To study this reaction, methods based on the exploration of the potential energy surface of FAD have been largely employed [30, 31, 32]. These studies have simulated the thermal catalytic reaction in gas‐phase [23, 27, 28, 29], in a solvent like water [22], for example, on small Pd clusters [25] and single atom‐Pd catalyst [24]. According to these studies, the reaction intermediate of the dehydrogenation route is the HCOO moiety which is adsorbed on the surface of Pd along with H.

The ability of plasmonic nanoparticles (NPs) to absorb and control light can greatly enhance the photocatalytic performances [33, 34, 35, 36, 37, 38, 39, 40]. This result may be explained in terms of the decay of localized surface plasmon resonances, that is, collective electron excitations which in turn decay rapidly in electron‐hole hot pairs [41, 42, 43, 44, 45, 46, 47, 48]. In plasmon‐mediated photocatalysis, one exploits the multicomponent nature of the experimental setup to enhance production rate and selectivity in reactions [49, 50, 51, 52, 53, 54, 55, 56, 57, 58, 59, 60]. The idea is to couple plasmonic nanostructures with (smaller) transition metal ones with well‐known catalytic properties. The combination of plasmonic and catalytic metal nanostructures in proximity, forming a strongly coupled “antenna reactor” complex [57, 61], is therefore a natural solution to obtain the best of the two components. The plasmonic nanoantenna is used to harvest light and directly enhance absorption in the nearby catalytically active reactor. Upon interaction with the external electromagnetic field, the plasmonic antenna induces an optical polarization in the reactor particle through its near field, thus driving a plasmon in the reactor. The plasmon decay produces hot carriers in the reactor, which in turn permits the exploration of new reaction pathways [44, 62].

In the context of the photocatalytic H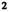 generation from FA [34], an example of antenna‐reactor setup is provided by Zheng et al. [63], with a tipped Au nanorod with Pd used to investigate the FAD. The observed hydrogen production rate matched levels typically achieved at high temperatures, with a distinct plasmonic effect playing a key role. In addition, the authors reported an increase in selectivity toward the dehydrogenation pathway.

Theory is essential to interpret experiments and elucidate the mechanisms driving plasmon‐mediated photocatalytic reactions [64, 65]. Since such mechanisms are based on ultrafast processes (the plasmon decay), to understand plasmonic effects on photocatalysis we must use time‐resolved methods in the femtosecond (fs) timescale [66, 67, 68, 69]. Building on the work in Reference [63]., here we focus on the effect of different Pd clusters and pulse frequencies on the photoinduced charge distribution of the target system. To study the FAD that produces CO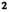 and H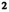, the chosen targets are the HCOO species and the hydrogen atom adsorbed on different Pd(111) clusters serving as catalytic centers. We used real‐time first‐principle methods based on the propagation of the time‐dependent Schrödinger equation (TDSE) for the electronic degrees of freedom interacting with an explicit pulse, at frozen nuclei [70]. In general, the importance of nuclear motion in plasmon decay in metal nanoclusters has been theoretically pointed out over the years [71, 72, 73, 74]. The goal is to first investigate the effect of plasmonic frequency on the reactor in the presence of HCOO and H: How does the reactor itself, without plasmon dynamics, react to the plasmon frequency? Indeed, the results of this work represent a preliminary but necessary study to simulate the dynamics of hot carriers in the full multiscale system. The next step, in future work, will be to consider the Au nanorod in time‐resolved simulations with an explicit pulse, to represent the experimental conditions in Reference [63]. We have also studied the photoinduced dynamics in the case of a tailored pulse which facilitates the FAD towards hydrogen production.

In Section 2, the theoretical framework is presented, while we collect the computational details in Section 3. The results are reported and discussed in Section 4, and the conclusions are given in Section 5.

## 2 Theory

Photoinduced electron dynamics is simulated by propagating the TDSE which is given in atomic units and length gauges by

(1) $$i\frac{d}{dt}\left|\Psi (t)\right\rangle =Ĥ(t)\left|\Psi (t)\right\rangle$$

$\left|\Psi (t)\right\rangle$ is the time‐dependent wavefunction while Ĥ(t) is the time‐dependent Hamiltonian, which is composed of a field‐free electronic Hamiltonian ${Ĥ}_{0}$ and a coupling term between the electric dipole operator $\hat{\mu }$ and the electric field ${\vec{E}}_{ext}(t)$ from and external pulse

(2) $$Ĥ(t)={Ĥ}_{0}-\vec{\hat{\mu }}\cdot {\vec{E}}_{ext}(t)$$

The time‐dependent |Ψ(t)⟩ is defined as a linear combination of the $N_{\text{states}}$ eigenstates of the field‐free Hamiltonian ${Ĥ}_{0}$

(3) $$\left|\Psi (t)\right\rangle =\overset{N_{states}-1}{\underset{M=0}{\sum }}C_{M}(t)\left|M\right\rangle$$

with $C_{M}(t)$ being the time‐dependent coefficients of the expansion and $\left|M\right\rangle$ is the Mth eigenstate of the system. In the space of these eigenstates, one can rewrite the TDSE as

(4) $$i\frac{d\text{C}(t)}{dt}=\text{H}(t)\text{C}(t)$$

where C(t) is the vector of the expansion coefficients and H(t) is the matrix representation at time t of the time‐dependent Hamiltonian, that is, ${\text{H}}_{LM}$ = $\left\langle L\right|Ĥ(t)\left|M\right\rangle$ which is diagonal for the field‐free part of the eigenenergies $E_{M}$ and contains the transition electric dipole moments between states,

(5) $$\left\langle L\right|Ĥ(t)\left|M\right\rangle =E_{M}\delta_{LM}-\underset{\gamma }{\sum }F_{\gamma }(t)\left\langle L\right|\vec{\hat{\mu }}\left|M\right\rangle$$

where γ=x,y,z indicates the three coordinates of the transition electric dipole moment.

To describe the excited states, we use approximated time‐dependent density functional theory eigenvectors with tight binding (TD‐DFT+TB) [75] within a configuration‐interaction singles ansatz [76], as implemented in the Amsterdam Modeling Suite (AMS) [77]:

(6) $$\left|M\right\rangle =\overset{occ}{\underset{i}{\sum }}\overset{vir}{\underset{a}{\sum }}d_{i,M}^{a}\left|\Phi_{i}^{a}\right\rangle$$

where $\left|\Phi_{i}^{a}\right\rangle$ is the singly excited Slater determinant with an electron promoted from the occupied molecular orbital (MO) i to the virtual one a, and $d_{i,M}^{a}$ are the amplitudes for the expansion of the excited state $\left|M\right\rangle$. In TD‐DFT+TB, a DFT ground‐state calculation provides the molecular orbitals for the subsequent linear‐response calculation with tight‐binding approximations [75].

The external electric field ${\vec{E}}_{ext}(t)$ is given by

(7) $${\vec{E}}_{ext}(t)={\vec{E}}_{max}\exp\left(-\frac{{\left(t-t_{0}\right)}^{2}}{2\sigma^{2}}\right)\sin(\omega t)$$

where $t_{0}$ is the center and σ the amplitude of the Gaussian envelope function, ${\vec{E}}_{max}$ is the maximum amplitude of the field, and ω is the pulse frequency.

Analysis of the electron dynamics is performed using the time‐dependent projected density of states (PDOS(t,ϵ)) [78], which is defined as the expectation of the value of the number operator $\hat{n}$ to the wavefunction $\left|\psi (t)\right\rangle$. Specifically, we compute the differential PDOS at time t (ΔPDOS), which refers to the initial condition at t=0

(8) $$\begin{array}{ll} & \Delta {\text{PDOS}}_{K}(t,\epsilon )\\ & \;=-\overset{occ}{\underset{i}{\sum }}w_{i}^{K}\text{Re}\left[\underset{M,L}{\sum }C_{L}^{*}(t)C_{M}(t)\overset{vir}{\underset{a}{\sum }}d_{i,L}^{a*}d_{i,M}^{a}\right]F_{\eta }(\epsilon -\epsilon_{i}) \end{array}$$

(9) $$\begin{array}{ll} & \;+\overset{vir}{\underset{a}{\sum }}w_{a}^{K}\text{Re}\left[\underset{M,L}{\sum }C_{L}^{*}(t)C_{M}(t)\overset{occ}{\underset{i}{\sum }}d_{i,L}^{a*}d_{i,M}^{a}\right]F_{\eta }(\epsilon -\epsilon_{i}) \end{array}$$

In Equation (9), $d_{i,M}^{a}$ ($d_{i,L}^{a}$) are the linear coefficients of the expansion for state $\left|M\right\rangle$ ($\left\langle L\right|$) and $F_{\eta }$ is a Lorentzian function centered on the MO energies $\epsilon_{i}$, with width η, used to obtain a smooth profile. Mulliken weights $w_{i}^{K}$ are used in the fragmentation of the studied system. Details are found in Reference [78]. The subscript/superscript K refers to the fragment, that is, the single atom or group of atoms, to which one computes the charge population.

The time‐dependent charge (electron and hole) population to the initial condition is defined as [64, 65]

(10) $$\begin{array}{ll} & \text{electron population}\\ & \;=\frac{1}{2}\int_{-\infty }^{+\infty }[\Delta {\text{PDOS}}_{K}(t,\epsilon )+|\Delta {\text{PDOS}}_{K}(t,\epsilon )|]d\epsilon \; \end{array}$$

and

(11) $$\begin{array}{ll} & \text{hole population}\\ & \;=\frac{1}{2}\int_{-\infty }^{+\infty }\left[\Delta {\text{PDOS}}_{K}(t,\epsilon )-|\Delta {\text{PDOS}}_{K}(t,\epsilon )|\right]d\epsilon \; \end{array}$$

## 3 Computational Details

**FIGURE 1:**
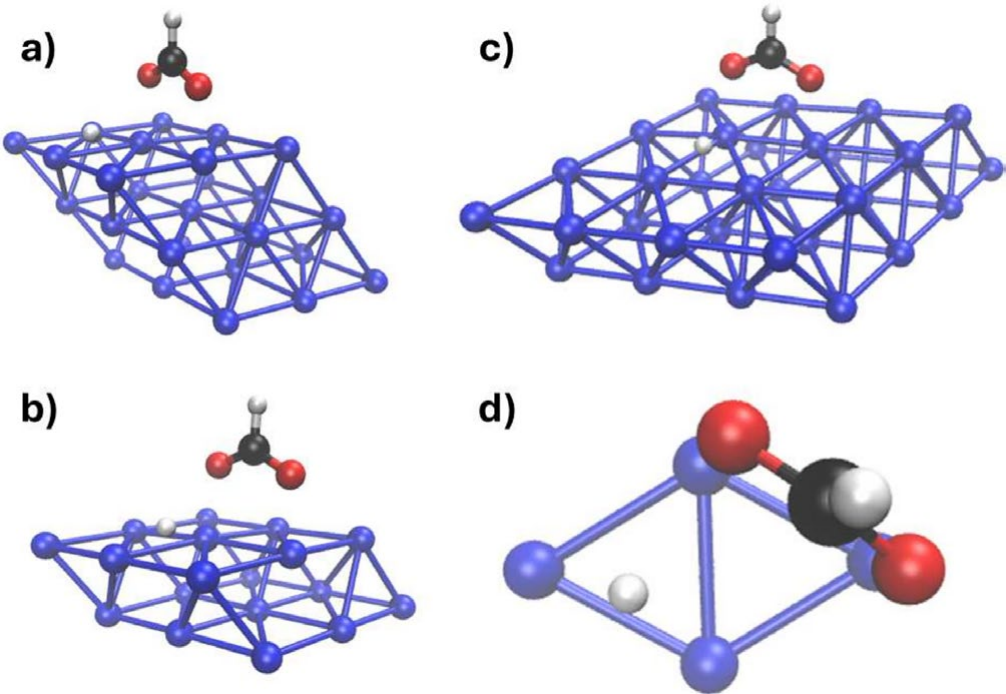
Structures analyzed and studied. HCOO and H are adsorbed of 3(3×3) (a), 2(3×3) (b), 2(4×4) (c), and Pd (111) surface. (d) Top view of the adsorption site of HCOO and H on the surface unit.

We studied the electron/hole dynamics for the three systems given in Figure 1: 3L3 with 27 Pd atoms (a), 2L4 with 32 Pd atoms (b), and 2L3 with 18 Pd atoms (c). The notation ALB refers to A layers and B×B Pd atoms per layer. Structures of the three systems in Figure 1 have been extracted from a geometry optimization of a slab of Pd(111) with HCOO and H adsorbed on top of it. All calculations were performed using the projector augmented wave method [79], as implemented in VASP software [80]. The PBE functional [81] with the van der Waals correction proposed by Grimme [82] was used for the geometry relaxation. Electron smearing of σ = 0.1 eV was used with the Methfessel–Paxton scheme [83]. The Brillouin zone was sampled by using the Monkhorst–Pack scheme, with an employed k‐mesh of 3×3×2 [84]. In addition, a plane‐wave basis set with a cutoff energy of 520 eV was used. A large vacuum of 14.5 Å was used to avoid spurious interactions. All structures were optimized until the Helmann–Feynman forces (that acting on each atom) were less than 0.01 eV/Å. The single unit cell is the one shown in Figure 1d). In the calculation, the first two layers have been left free to reorganize, while the third layer was left in the crystalline position of the Pd(111) surface. From this initial slab, we extracted three finite systems: A cluster with three layers (3L3), two clusters with two layers, that is, 2(3×3) (2L3) and 2(4×4) (2L4) (Figure 1). The geometries obtained after the optimization are in good agreement with the literature reference [85] (see Table S1 in Supporting Information (SI)). Cartesian coordinates are also reported in SI. In all cases, the system, that is, Pd layers + HCOO + H, is neutral.

**FIGURE 2:**
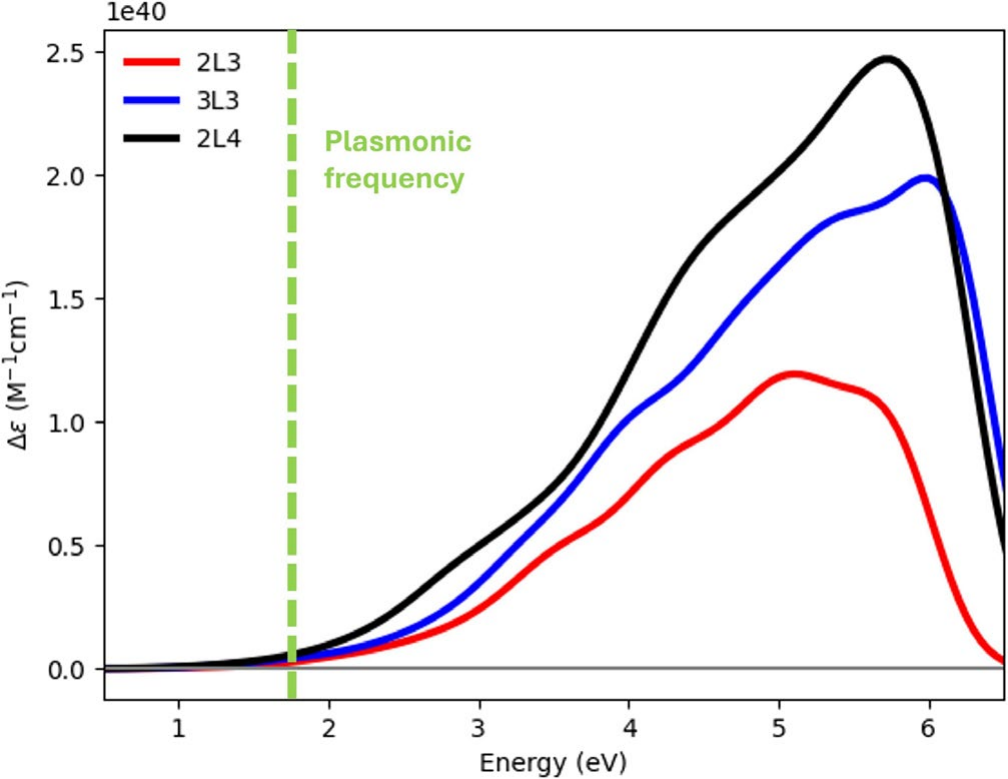
Absorption spectra of the 2L3, 3L3, and 2L4 systems. The green dashed line corresponds to the plasmonic frequency of the Au nanorod.

Real‐time calculations were carried out using the WaveT package [86], interfaced with AMS for extracting electric transition dipole moments [66, 70, 78]. Excitation energies and transition dipole moments, calculated using TD‐DFT+TB [75] with the RPBE functional [87] combined with Grimme corrections, and DZ basis set for all three systems, served as inputs for real‐time propagation. For each system, 100‐fs dynamics were simulated with a time‐step δt of 1 as. Wavefunction expansion in Equation (3) includes 1,808, 4,000, and 5,500 excited states for the 2L3, 3L3, and 2L4 systems, respectively, covering excitations up to 6 eV for 2L3, 6.4 eV for 3L3 and 6.3 eV for 2L4. In Figure 2 the absorption spectra of the three systems along with the plasmonic frequency of a model Au nanorod (13.2 nm of length, 3 nm of diameter) is reported (Figure S1 of the SI). The Au nanorod is modeled using the polarizable continuum model; details are found in References [66, 86].

The applied external field ${\vec{E}}_{ext}(t)$ of Equation (7) has a peak intensity of $10^{2}$ W/cm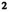 and a full‐width half maximum (FWHM) of 21 fs for the Gaussian envelope. This choice for FWHM is based on the fact that an ultrafast pulse simulates approximately the effect of a CW light with a coherence length of few μm, as typically used in the experiments [65]. In all cases, the pulse is linearly polarized as perpendicular to the Pd layers. Two frequencies were selected for the electronic dynamics simulation: The plasmonic frequency (P1) of the Au nanorod (length of 13.2 nm, diameter of 3 nm) at 681 nm (1.82 eV, see Figure 1 in SI) and a frequency (P2, for the 3L3 system only) that populates the anti‐bonding MOs facilitating the C–H bond breaking. The P1 frequency has been computed by means of the boundary element method (BEM) coupled to the polarizable continuum model [86]; details of the BEM calculation are given in the caption of Figure S1 of the SI.

In all the cases, the system is initially in the ground state, that is, $|C_{0}(0){|}^{2}=1$. Nuclei were kept frozen along the dynamics.

## 4 Results and Discussion

The goal is not to characterize the time‐evolution of a plasmonic response, but simply to observe the effect of the plasmonic frequency of a gold nanorod (the P1 frequency), which has not been explicitly included in the simulations, on the photoinduced electron dynamics in Pd clusters interacting with HCOO and H species.

Electron‐dynamics simulations were performed on the systems of Figure 1, which include the intermediates, that is, HCOO and H, of the dehydrogenation reaction of FA. 2L3, 2L4 and 3L3 geometrical features closely match those reported in literature [27, 85]. Specifically, the bridge‐like adsorption configuration of HCOO on the Pd surface and the preference of H to adsorb at the hcp site are consistent with the results of published computational works [27, 85]. HCOO was identified to be the main intermediate of the reaction pathway [22, 27, 85], ultimately leading to the formation of CO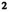 and H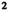. The rationale behind the choice of 2L3, 2L4, and 3L3 clusters is based on exploring the number of layers and Pd atoms for converged results in terms of electron dynamics in a finite‐size system.

Starting from these geometries, TD‐DFT+TB calculations were carried out using AMS as a preliminary step before performing the electron/hole dynamics by means of WaveT [66, 68]. Post‐processing tools [64, 65, 78] allow us to analyze the electron/hole dynamics in terms of charge transfer processes among the various components of the system. Quantities such as ΔPDOS [65, 78] (Equation (9)) and its energy integrals (Equations (10) and (11)), along with the evolution of electron and hole populations over time, were computed to describe the dynamics of the target systems.

**FIGURE 3:**
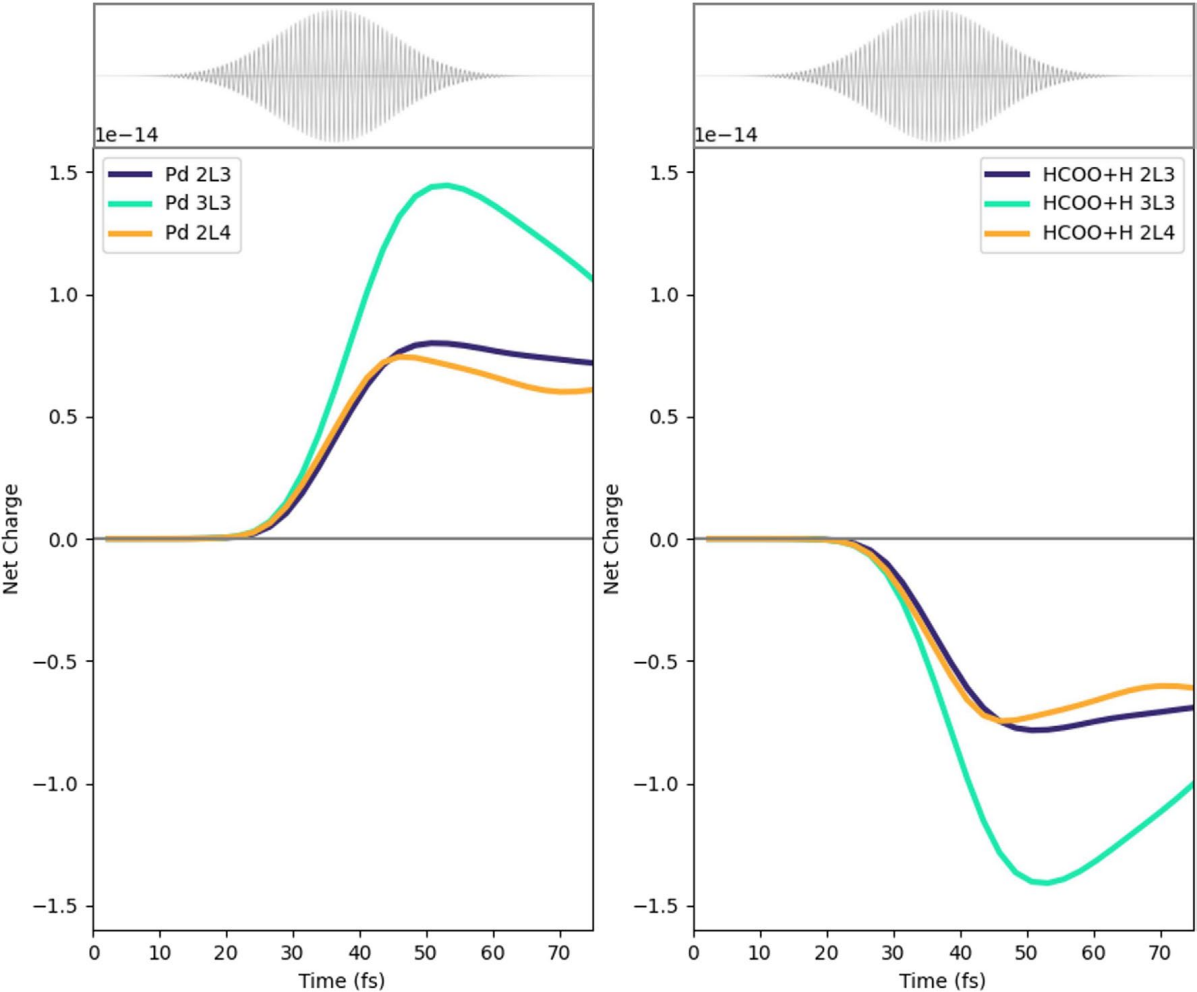
Left panel: Time‐evolution of the net charge of the Pd cluster for the three analyzed systems (2L3, 3L3, and 2L4) with the P1 pulse. Right panel: Time‐evolution of the net charge of the HCOO species for the three analyzed systems with the P1 pulse.

In Figure 3, the time‐evolution of the net charge of the Pd cluster (left panel) and of the HCOO+H species (right panel) is shown for 2L3, 2L4, and 3L3. The P1 pulse, that is, the pulse with the plasmonic frequency, is also reported as a reference. The net charge is defined as the difference between the hole (Equation (11), K = Pd cluster or HCOO+H) and electron (Equation (10)) population: A positive (negative) value indicates a defect (accumulation) of electrons.

A net electron injection into HCOO and H is observed regardless of the specific shape of the Pd cluster. Charge separation is more efficient with 3L3, while 2L3 and 2L4 are characterized by a very similar profile up to around 45 fs, after the maximum of the pulse. At larger times, charge separation in 2L4 is smaller than that in 2L3. Intriguingly, the amount of charge separation does not follow simply the number of Pd atoms, but it rather seems to be a function of the number of layers and, in general, of the topology of the Pd cluster.

Of course, the sum of the net charge on Pd, HCOO, and H is zero, because no other source or sink of charge is present in the simulations.

From Figure 3, one observes that the pulse maximum is at around 35 fs, while it ends at around 60 fs. In the next subsections, we report and discuss the results of the dynamics in terms of the charge of defined fragments, emphasizing differences and similarities between 2L3, 2L4, and 3L3. Plus, for 3L3 only, we also show the charge population induced by the P2 pulse.

### 4.1 2L3 and 2L4

From now on, the charge population is represented as a percentage, which is calculated by dividing the value of the electron/hole population of the fragment over time by the maximum value of the electron/hole population of the full system (Pd cluster + HCOO + H); the percent net charge is then calculated from the percent populations of negative and positive charge. If not explicitly stated, the P1 pulse was used in the calculations.

**FIGURE 4:**
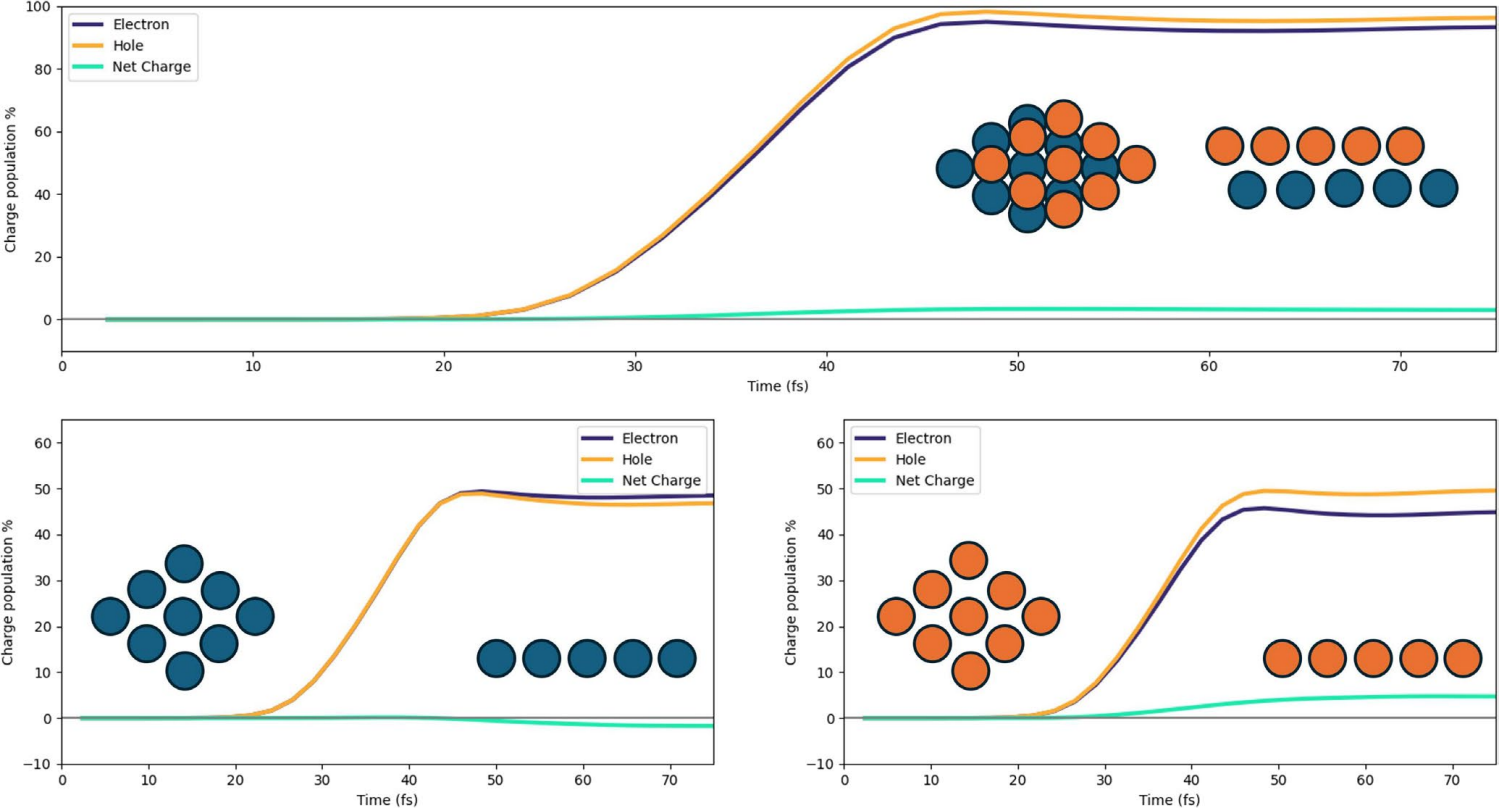
Upper panel: Time‐evolution of the photoinduced charge populations (electron, hole, and net) of the full Pd cluster in the 2L3 system with P1 pulse. Bottom panels: Time‐evolution of the photoinduced charge populations (electron, hole, and net) of the bottom layer (left) and upper layer (right).

The three panels of Figure 4 report the time‐evolution of the photoinduced charge population of Pd atoms, for the 2L3 system. P1 pulse has been used. In the top panel, the results are displayed for the full Pd cluster, whereas in the bottom panels, the data regarding the lower (dark blue) and upper (orange) layers are displayed. The lower layer is slightly negatively charged at times at which the pulse is already very small or zero, indicating that it is not a direct photoinduced process. The upper layer has a net positive charge that arises between 30 and 40 fs; moreover, the net charge is also larger than the one in the lower layer. These two findings give evidence that electronic charge has been directly transferred from the upper PD layer to the adsorbed species due to the pulse. A net positive charge in the Pd cluster is also observed in Figure S2 of the SI for the 2L4 case. Here, both layers are slightly positively charged according to a direct photoinduced mechanism [65]. Increasing the size of the Pd layer appears to have minimal impact on the overall electron and hole populations of the system, with only minor variations observed in the behavior of single layers.

**FIGURE 5:**
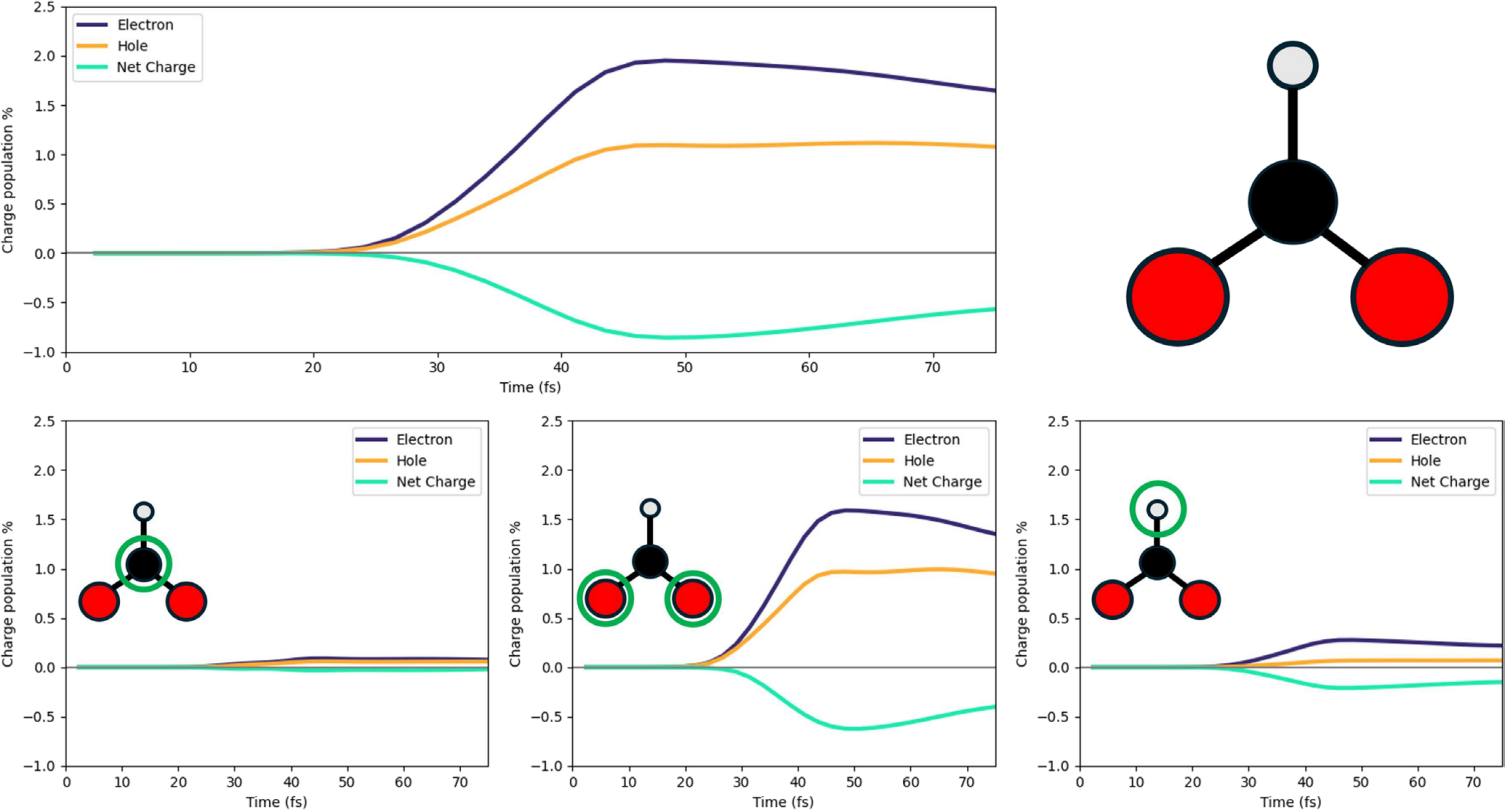
Upper panel: Time‐evolution of the photoinduced charge populations (electron, hole, and net) of the HCOO fragment in the 2L3 system with P1 pulse. Bottom panels: On the left, the time‐evolution of the photoinduced charge populations (electron, hole, and net) of the carbon atom; in the middle, the same for the oxygen atoms; on the right, the same for the hydrogen atom of HCOO.

In Figure 5, the charge populations of the HCOO moiety are reported. The top panel displays the electron and hole population of the entire HCOO fragment, revealing an excess of electrons, that is, the net charge is negative. This negative charge arises from an electron injection process, with electrons being transferred from the upper Pd layer to the molecular species, as already stated. The bottom three panels of Figure 5describe the charge population of the carbon atom (left), two oxygen atoms (middle), and hydrogen (right) of HCOO. The negative charge in HCOO is mainly concentrated on the two oxygen atoms, which interact with the positively charged Pd atoms of the upper layer. A similar result is found for 2L4 (Figure S3 of the SI), even though the percentage of negative charge on C and H is larger in this case.

The HCOO charge populations observed in 2L3 and 2L4 can also be rationalized by inspecting the ΔPDOS (Equation (9)) of the fragment before (t=0) and after (at around 65 fs) the pulse (Figures S4 and S5 of the SI, respectively). This quantity shows how the populations of the molecular orbitals are changed at a given time to the initial orbital population, that is, t=0. Negative peaks indicate the depopulation of the corresponding orbital, whereas the positive peak indicates an increase in the population. ΔPDOS of the HCOO before and after the pulse is reported in Figures S4 and S5 of the SI along with the occupied and virtual molecular orbitals that are involved in the excitation. The occupied ones are mostly centered on the Pd atoms (indicating a strong interaction between Pd atoms and adsorbed species), whereas the virtual ones also involve HCOO (especially the oxygen atoms) and H species. This is an ulterior proof of the fact that the P1 pulse is moving electrons from the Pd layers to the molecular species.

Of course, we are interested in the charge population on HCOO from a photocatalytic point of view, but still, we also analyzed the time‐evolution of the photoinduced charge on the hydrogen atom. For all systems, the net charge in H is about two orders of magnitude larger than in HCOO. The time‐dependent electron and hole populations of the adsorbed H atom for 2L3 and 2L4 are reported in Figures S6 and S7 of the SI, respectively. In both cases, the H atom acquires a negative net charge.

**FIGURE 6:**
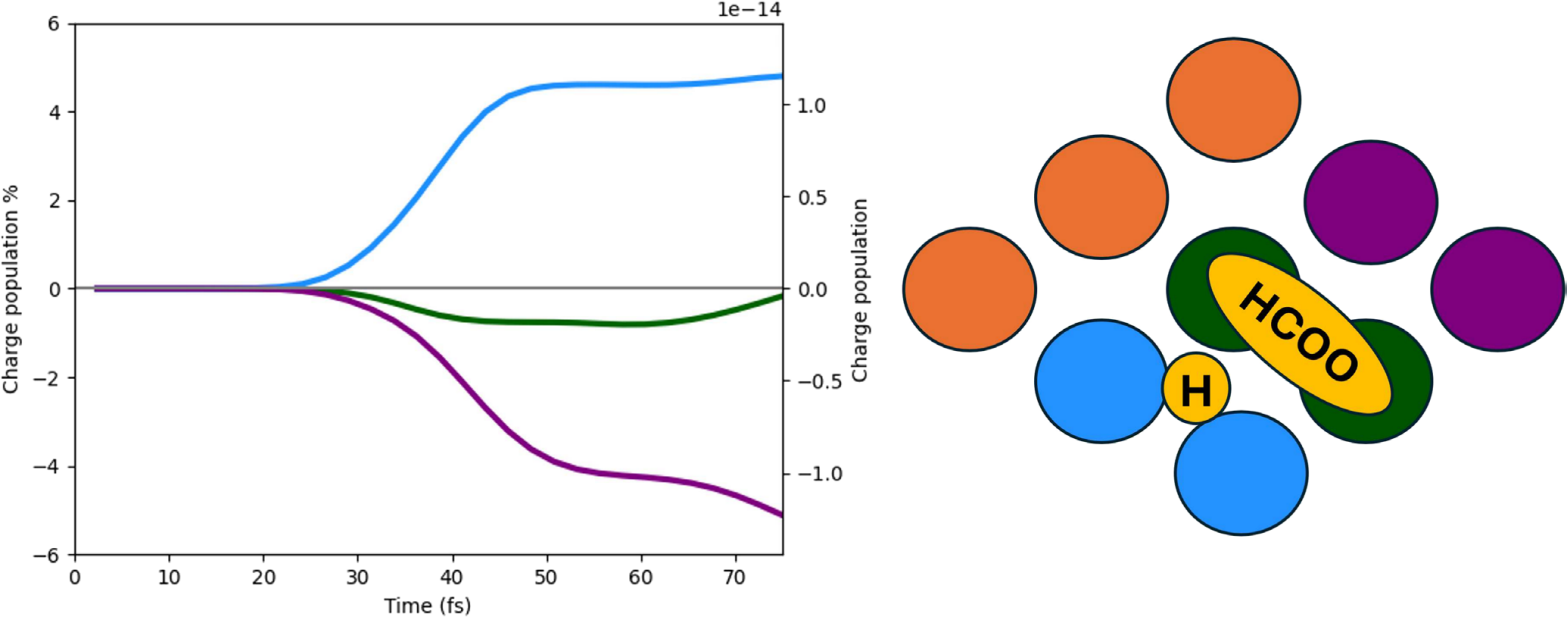
Time‐evolution of the photoinduced net charge of the Pd atoms of the upper layer interacting with the molecular species in the 2L3 system with P1 pulse.

Another piece of information that we can extract from real‐time simulations is how the surface charges of the slab are modified due to the interaction with the pulse. Indeed, surface‐charge heterogeneity is considered one of the main factors determining the enhancement observed in plasmon‐assisted photocatalysis [63]. In Figure 6, the net charge population of the Pd atoms in proximity to the HCOO and H is reported. Pd atoms that interact with HCOO are reported in green, in blue the ones that interact with H, and in purple two other neighbor Pd atoms. The Pd atoms belong to the upper layer. The Pd pair which interacts with HCOO and the close purple pair tends to acquire a net negative charge, whereas the one interacting with H is positively charged. Different slopes in the time profile suggest the presence of different mechanisms of charge injection. The same analysis has been performed for 2L4, as shown in Figure S8 of the SI. Differently from 2L3, the only spot negatively charged is given by the two Pd atoms interacting with HCOO. Also, the absolute value of the charges of the 2L4 system is smaller than the ones of the 2L3 case.

In conclusion, we can infer that increasing the size of the Pd layer does not affect the type of charge injection into HCOO. However, the internal dynamics of the Pd cluster have been observed to depend on the number of Pd atoms per layer.

### 4.2 3L3

**FIGURE 7:**
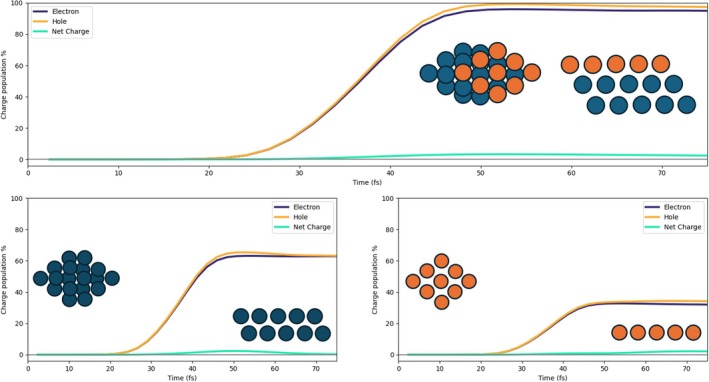
Upper panel: Time‐evolution of the photoinduced charge populations (electron, hole, and net) of the Pd cluster in the 3L3 system with P1 pulse. Bottom panel: Time‐evolution of the photoinduced charge populations (electron, hole, and net) of the two lower Pd layers (left) and upper one (right).

**FIGURE 8:**
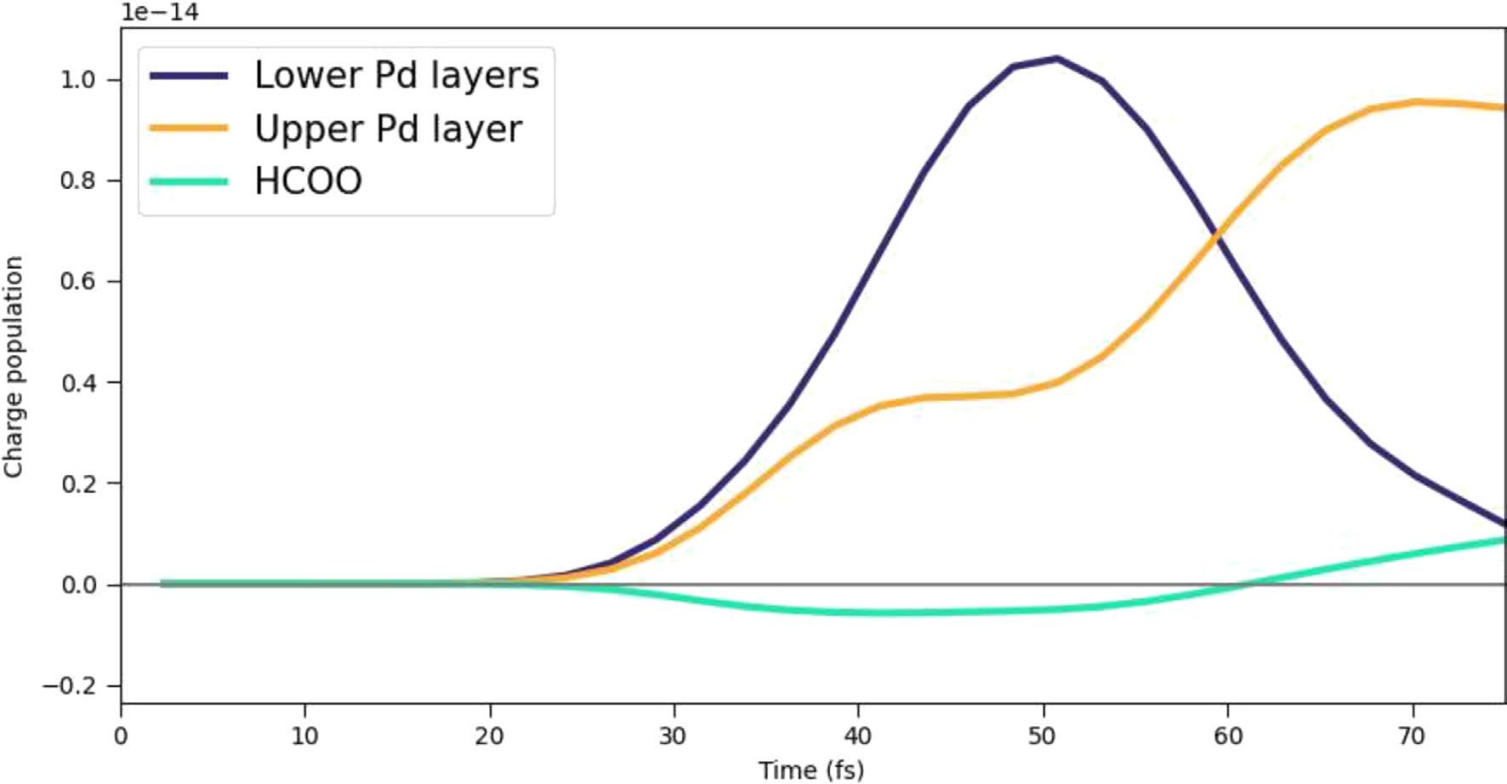
Time‐evolution of the net charge of the lower Pd layers, upper Pd layer, and HCOO.

In the 3L3 system, three Pd layers are explicitly considered. In Figure 7, the charge populations of the Pd cluster are reported, as we did for 2L3 and 2L4. Also, in this case, a net positive charge, generated by a direct photoinduced process, is present on the fragment due to the transfer of electrons from Pd to the molecular species adsorbed on top of the upper layer. The two bottom panels of Figure 7show the charge population of the upper layer (right) and two lower layers (left). In the left bottom panel, we can observe that the excess of hole population decreases after 60 fs, and a consequent hole excess on the upper layer tends to arise, indicating an indirect, that is, not mediated by light, charge transfer [64, 65]. This indirect charge‐transfer mechanism also involves HCOO. We compare in Figure 8 the net charge of the lower Pd layers, upper Pd layer, and HCOO, in absolute terms. The decrease of the net charge of the lower Pd layers starting at 50 fs, that is, at the tail of the pulse, coincides with an increase of the net charge of the lower Pd layer and with the delayed change of sign of the HCOO net charge, occurring at 60 fs. These data are interpreted as a hole transfer from the lower Pd layers to the upper Pd layer and HCOO.

**FIGURE 9:**
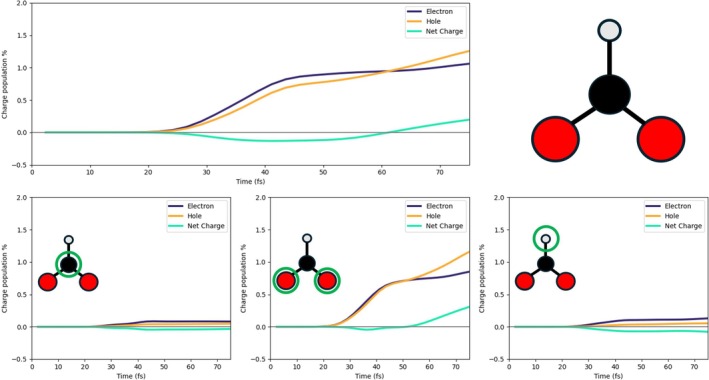
Upper panel: Time‐evolution of the photoinduced charge populations (electron, hole, and net) of the HCOO fragment in the 3L3 system with P1 pulse. Bottom panels: On the left, the time‐evolution of the photoinduced charge populations (electron, hole, and net) of the carbon atom; in the middle, the same for the oxygen atoms; on the right, the same for the hydrogen atom of HCOO.

The net charge of HCOO is also given as a percentage in the upper panel of Figure 9. Atoms of HCOO involved in the indirect charge transfer, and therefore in the sign flip of the net charge, are the O atoms, which strongly interact with the Pd ones of the upper layer, as argued from the lower panels of Figure 9. Indeed, while C and H atoms of HCOO acquire a small negative charge upon irradiation holding along the dynamics, O atoms are characterized by a transient negative charge between 30 and 40 fs approximately, and then a hole injection on them is observed.

Analysis of the ΔPDOS and the molecular orbitals involved in the dynamics (Figure S9 of the SI) allows one to further confirm this interpretation. In this case, the ΔPDOS at 45.97 fs has been reported as well, together with the data at 65.50 fs and those at the beginning of the dynamics (t=0). In this way, one can observe changes in the orbitals occupation before (t<50 fs) and after (t>50 fs) the hole injection. Table S2 in SI reports the values of the ratio (65.50 fs)/45.97 fs) of the absolute value of ΔPDOS peaks. These ratio values are around 1, that is, no change in the dynamics occurs before and after 50 fs, except for the occupied orbitals labeled “4” and “5”. These two orbitals, which maintain a strong oxygen character, become increasingly depopulated at 65.50 fs, with a correspondingly higher value of the ratio (2.09 for “4”, 1.63 for “5”). This indicates that the depopulation of these orbitals does not contribute to the population of the virtual orbitals projected on the HCOO fragment.

**FIGURE 10:**
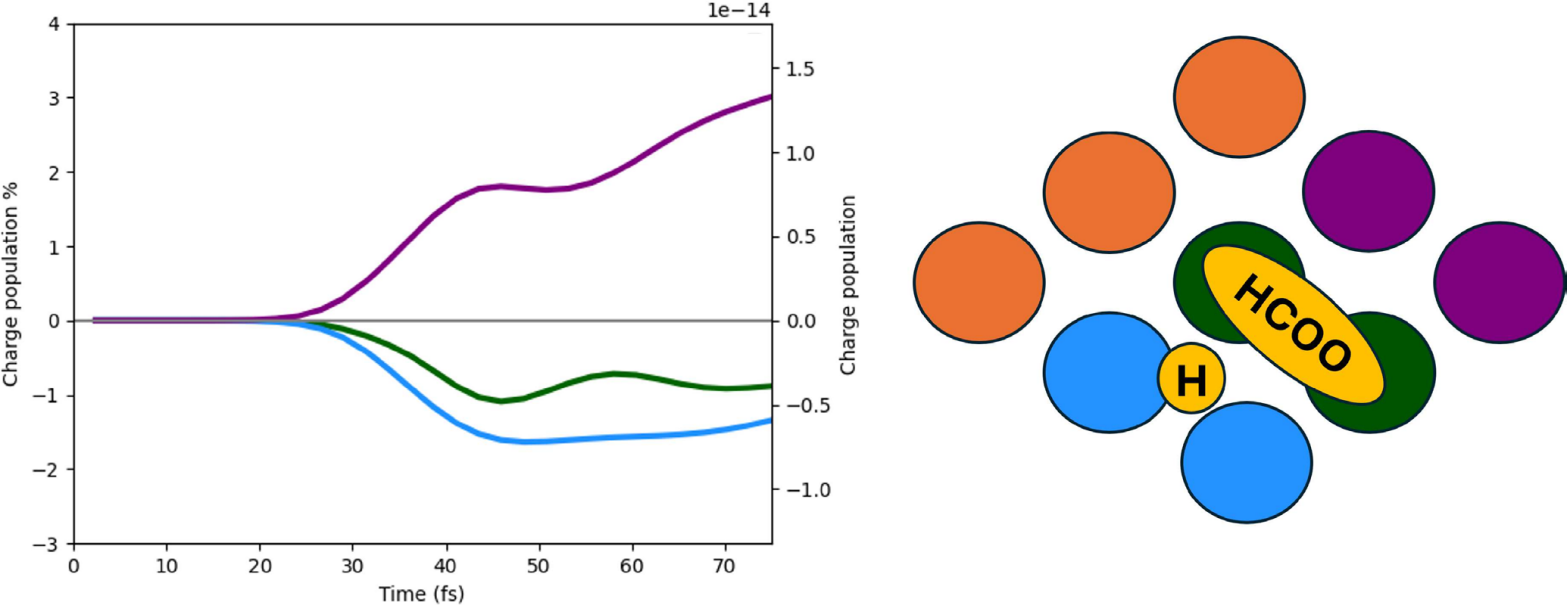
Time‐evolution of the photoinduced charge populations (electron, hole, and hole) of the Pd atoms of the upper layer interacting with the molecular species in the 3L3 system with P1 pulse. Green: Pd pairs interacting with HCOO. Blue: Pd pairs interacting with H. Purple: Pd pairs not interacting with any molecular species.

Charge separation in the upper layer of Pd atoms also occurs in the 3L3 case, as shown in Figure 10. The pairs of Pd atoms interacting with the adsorbed species become negatively charged. Interestingly, this analysis also gives evidence of the indirect mechanism at times greater than 50 fs, with a change in the time profile of the pair interacting directly with HCOO and the adjacent pair without adsorbates.

The evolution of the electron, hole, and net charge populations of the adsorbed H atom is reported in Figure S10 of the SI. A negative charge is accumulated on H, in greater amounts than in the case of 2L3 and 2L4.

In percentage, the photoinduced charge population in HCOO is greater in the 2L3 case, as evident from Figures 5 and 9, and Figure S3 of the SI. This result can be interpreted in terms of the greater tendency of electrons to escape when the number of Pd atoms is reduced, as precisely in the 2L3 case.

It is interesting to point out that in light‐off conditions the system is already polarized, with HCOO being negatively charged, as shown in Table S3 of the SI; this result is obtained for each of the systems studied.

The P1 pulse frequency was selected since it corresponds to the excitation energy of the plasmon of a gold nanorod in Figure S1 in SI. All the analysis conducted so far thus represents a preparatory step to the study of the complete system, in the presence of the Au nanorod, which will take place in future work. As a main message, we have verified that regardless of the type of Pd cluster, an injection of electrons to HCOO is verified; this will allow us greater flexibility of reactor choice in modeling the antenna‐reactor complex, with the understanding that differences between 2L3, 2L4 and 3L3 in photoinduced processes have been found and discussed here.

Using the P1 pulse thus allows adherence to the experimental work [63]. What if we used instead a pulse frequency that facilitates the FA dehydrogenation by populating the proper antibonding molecular orbitals? From the output of the TD‐DFT+TB calculation, we can determine the character of the electronic excitations in terms of transition among orbitals. For the P2 pulse we selected a frequency that populates orbitals that facilitate the FA dehydrogenation. In our case, we selected the frequency of an excitation that populates anti‐bonding orbitals localized on the C‐H bond of HCOO, which leads to hydrogen formation. The selected wavelength for the P2 pulse for the 3L3 system is 198 nm (6.26 eV).

A net positive charge is found in the Pd cluster, as reported in Figure S11 of the SI, even though the upper layer is negatively charged. As expected, tailoring the pulse with the proper frequency maximizes the electron population on HCOO, which is now in percentage much larger than that in P1‐based dynamics, as shown in Figure S12 of the SI. In particular, we can notice that the electronic population is accumulated on C and H atoms of HCOO, leaving O atoms with a positive charge, which in turn is consistent with the negative net charge in the upper Pd layer. All these processes can be considered as a photoinduced direct mechanism; the indirect charge transfer is therefore suppressed with a “resonant” light‐matter interaction. It is worth noting that only the frequency P1, and frequencies that deviate slightly from P1, will be considered in the work that will contain the coupling with the gold nanorod, to verify the possible enhanced production rate, as reported experimentally. So, the use of P2 frequency, which is not experimentally explored, is limited to this work only.

**FIGURE 11:**
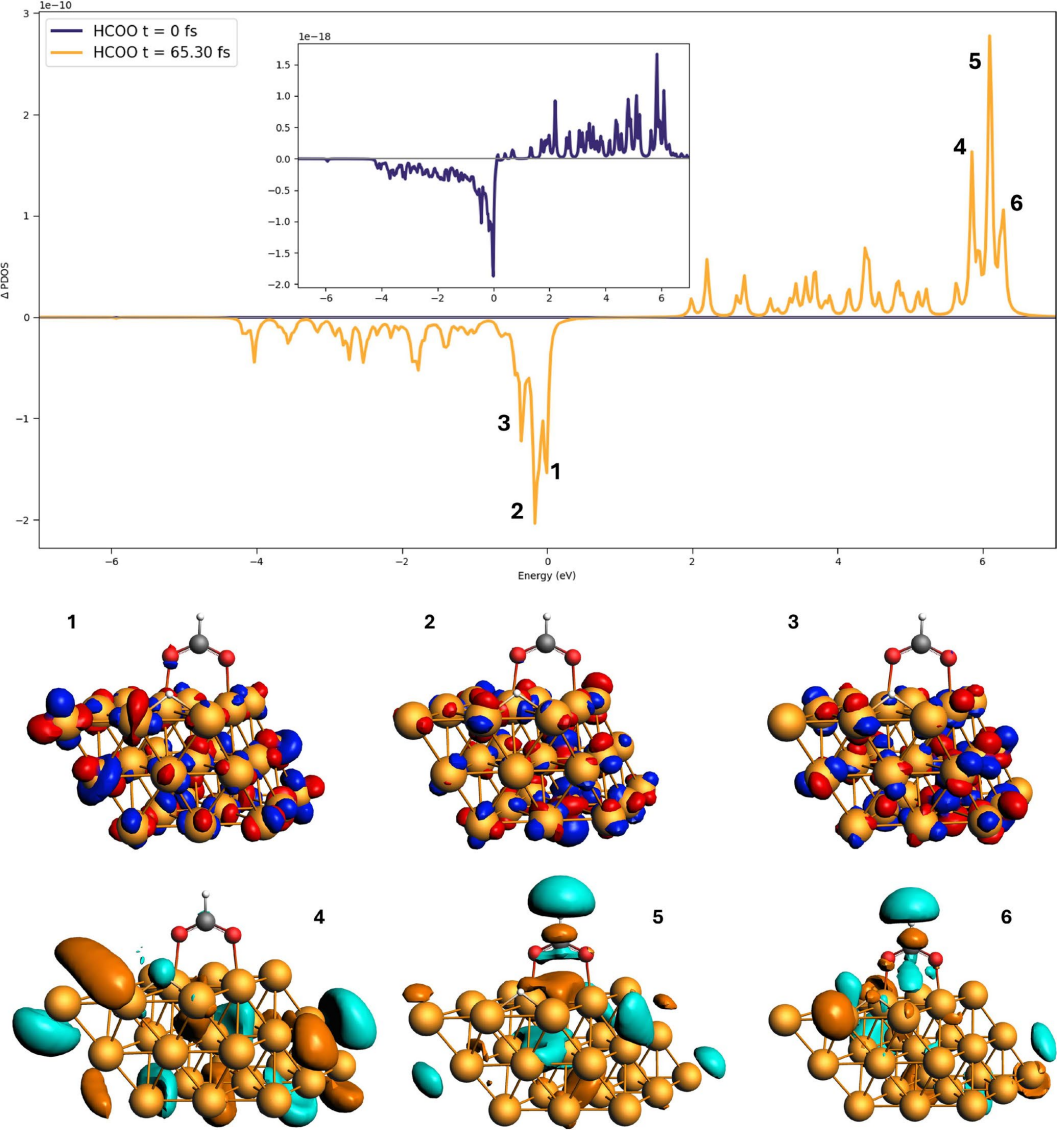
ΔPDOS of the HCOO fragment at t=0 (blue line) and t=65.30 fs (orange line) for the 3L3 system with the P2 pulse. The occupied and virtual orbitals involved in this excitation are also reported.

In Figure 11, we reported the ΔPDOS value before (t=0 fs) and after (t=65.30 fs) the laser peak for the 3L3 system. The orbitals which are getting depopulated are HOMO (“1”) or close to it (“2” and “3”), and they are mostly localized on the Pd cluster and the oxygen atoms of the HCOO fragment. The virtual orbitals that are getting populated instead are mostly localized on the HCOO fragment and they are mostly characterized by an antibonding character for the C‐H bond (orbitals “5” and “6”). In summary, the chosen pulse allows the transfer of electrons from the Pd cluster to the molecular moiety which, in this case, directly aids the catalytic process which eventually leads to the C‐H bond breaking. In Figures S13, S14, and S15 of the SI, we also present the ground‐state PDOSs of the Pd layers and HCOO for 2L3, 3L3, and 2L4. They share a similar general profile; some appreciable differences are found in the relative intensity of the peaks around −6 and −6.5 eV. The first peak corresponds to a π‐bonding orbital of the O‐C‐O bond and a σ‐bonding orbital of the Pd‐H orbital. The second peak presents a similar character, with a smaller degree of hybridization between the molecular moiety and Pd atoms. Moreover, the Pd PDOS of 3L3 shows a maximum at the HOMO energy, at variance with what occurs with 2L3 and 2L4.

To verify the effect of the chosen methodology on the results, we have also carried out full TDDFT calculations with RPBE and B3LYP functionals for 2L3, using the same basis set and Grimme corrections of the TD‐DFT+TB calculations. The TD‐DFT+TB/RPBE, TDDFT/RPBE, and TDDFT/B3LYP spectra are collected in Figure S16 of the SI: A very good agreement is observed, thus justifying the use of TD‐DFT+TB/RPBE. Moreover, electron dynamics based on TDDFT/B3LYP have been generated and analyzed: In Figures S17 and S18 of the SI, we report the time‐evolution of the charge population for the Pd layers and HCOO, respectively. Also in this case, a net electron injection into HCOO is predicted, according to the TD‐DFT+TB findings: A difference at large times (>50 fs), where modulation of the HCOO negative charge is observed, perhaps due to an indirect charge transfer, which is not present in TD‐DFT+TB/RPE data.

In all the studied systems, finite‐size effects are present, especially observed in the virtual molecular orbitals characterized, in some cases, by density at the border of the Pd layers.

Moreover, it is important to note that all results presented in this article were obtained with coherent frozen‐nuclei dynamics. More realistic modeling, especially at long times, in which nuclear motion is assumed to have been triggered, would therefore have to take into account environmental effects on electronic degrees of freedom. As we observe in Reference [64]. for the CHO moiety interacting with Rh layers, the effect of the environment in terms of electronic relaxation and dephasing does not affect the sign of the charge injection, thus justifying the use of closed electron dynamics to investigate the photoinduced charge transfer, as we did in this work. One can effectively model the non‐radiative decay by using the energy‐gap law [64].

## 5 Conclusions

In this study, we investigated the electron/hole dynamics of the HCOO moiety and the hydrogen atom, as intermediates in the FAD reaction, in the presence of Pd clusters of different sizes and layer configurations (2L3, 3L3, and 2L4). We used a time‐resolved approach based on solving TDSE in the space of DFT‐TB pseudo‐eigenstates [76, 88, 89, 90, 91, 92, 93].

The main goal was to evaluate how the choice of the catalytic cluster affects the dynamics, using mainly the P1 pulse, which is resonant with the plasmonic transition of an Au nanorod (not explicitly included in the TDSE calculations). To achieve this, we analyzed the time‐evolution of charge populations by means of ΔPDOS and its energy integral, focusing on the Pd layers, on HCOO as a whole and its atoms, and the adsorbed H. For all the systems, we observed a net transfer of negative charge from the Pd layers to HCOO and H. In the case of 2L3 and 2L4, the transferred charge is mainly localized on the oxygen atoms of HCOO, according to a photoinduced direct mechanism. In contrast, the 3L3 system exhibited much more complex dynamics: The direct process is followed by an indirect one, starting at around 50 fs, which provides a hole injection into HCOO.

Calculations also show an inhomogeneous charge redistribution in the upper Pd layer upon irradiation, which depends on the specific Pd cluster.

We also studied for 3L3 the effect of a pulse tailored to drive the reaction toward dehydrogenation, which consists of populating antibonding orbitals located on the C‐H bond of HCOO; C and H atoms of HCOO are indeed characterized by a large photoinduced electron population. In this case, oxygen atoms are positively charged while the upper Pd layer is negatively charged.

As a next step, we plan to include the Au nanorod in the model, to fill the gap with the experimental setup [63]. This will allow us to rationalize the enhancement observed in the plasmon‐assisted photocatalytic process.

## Conflicts of Interest

The authors declare no conflicts of interest.

## Supporting information




**Data S1.** Cartesian coordinates for 2L3, 2L4 and 3L3. The absorption spectrum of the Au nanorod. Results for 2L4. Charge populations for the adsorbed H atom. **ΔPDOS**. Ground‐state Mulliken charges of HCOO and H in 2L3, 3L3 and 2L4. Initial PDOS. Absorption spectra of 2L3 with TDDFT/RPBE and TDDFT/B3LYP. Time‐dependent charge population of the Pd layers and HCOO for 2L3 using TDDFT/B3LYP.

## Data Availability

The data that support the findings of this study are available from the corresponding author upon reasonable request.
