# Supplementary material for: Study of the Photoinduced Charge Injection in the Reaction Intermediate of the Dehydrogenation of Formic Acid on Palladium
Source: J Comput Chem. 2025 Mar 26;46(8):10.1002/jcc.70087. doi: 10.1002/jcc.70087 (PMC11938336; doi:10.1002/jcc.70087)
Supplement: Supplementary file 1 — Data S1. Cartesian coordinates for 2L3, 2L4 and 3L3. The absorption spectrum of the Au nanorod. Results for 2L4. Charge populations for the adsorbed H atom. ΔPDOS. Ground‐state Mulliken charges of HCOO and H in 2L3, 3L3 and 2L4. Initial PDOS. Absorption spectra of 2L3 with TDDFT/RPBE and TDDFT/B3LYP. Time‐dependent charge population of the Pd layers and HCOO for 2L3 using TDDFT/B3LYP. [file JCC-46-0-s001.pdf]

# Supporting Information of "Study of the photoinduced charge injection in the reaction intermediate of the dehydrogenation of formic acid on palladium"

L. Biancorosso[1] and E. Coccia[1, a)]

*Dipartimento di Scienze Chimiche e Farmaceutiche, Università di Trieste, via L. Giorgieri 1, 34127, Trieste, Italy*

(Dated: 6 March 2025)

[a)]Electronic mail: ecoccia@units.it

**2L3 Cartesian Coordinates (Å)**

Pd 20.18687 5.43823 2.29461

Pd 21.52310 7.77654 2.33418

Pd 22.87753 10.10346 2.31024

Pd 14.79856 5.44551 2.29707

Pd 16.14919 7.77636 2.32525

Pd 17.48698 10.10152 2.32705

Pd 17.48831 5.43919 2.29520

Pd 18.83717 7.77355 2.31898

Pd 20.18622 10.09607 2.34379

Pd 20.18573 8.53739 4.72623

Pd 21.56223 10.89762 4.64912

Pd 18.84032 6.21292 4.58033

Pd 22.87430 8.55204 4.61971

Pd 24.21889 10.89466 4.62662

Pd 21.53419 6.22063 4.61695

Pd 17.47678 8.55372 4.66778

Pd 18.81765 10.90645 4.63550

Pd 16.14256 6.21964 4.60739

C 18.72573 8.30817 7.35710

O 17.60863 8.50129 6.77983

O 19.88217 8.17731 6.85923

H 18.66924 8.24703 8.46262

H 20.19139 10.38844 5.55268

**3L3 Cartesian Coordinates (Å)**

Pd 20.18397 6.99194 0.00000

Pd 21.52957 9.32259 0.00000

Pd 18.83837 4.66129 0.00000

Pd 14.80158 6.99194 0.00000

Pd 16.14717 9.32259 0.00000

Pd 13.45598 4.66129 0.00000

Pd 17.49278 6.99194 0.00000

Pd 18.83837 9.32259 0.00000

Pd 16.14718 4.66129 0.00000

Pd 20.18687 5.43823 2.29461

Pd 21.52310 7.77654 2.33418

Pd 22.87753 10.10346 2.31024

Pd 14.79856 5.44551 2.29707

Pd 16.14919 7.77636 2.32525

Pd 17.48698 10.10152 2.32705

Pd 17.48831 5.43919 2.29520

Pd 18.83717 7.77355 2.31898

Pd 20.18622 10.09607 2.34379

Pd 20.18573 8.53739 4.72623

Pd 21.56223 10.89762 4.64912

Pd 18.84032 6.21292 4.58033

Pd 22.87430 8.55204 4.61971

Pd 24.21889 10.89466 4.62662

Pd 21.53419 6.22063 4.61695

Pd 17.47678 8.55372 4.66778

Pd 18.81765 10.90645 4.63550

Pd 16.14256 6.21964 4.60739

C 18.72573 8.30817 7.35710

O 17.60863 8.50129 6.77983

O 19.88217 8.17731 6.85923

H 18.66924 8.24703 8.46262

H 20.19139 10.38844 5.55268

**2L4 Cartesian Coordinates**

Pd 12.113285 5.438224 2.294608

Pd 16.150080 12.430161 2.294608

Pd 20.186874 5.438225 2.294608

Pd 24.223669 12.430162 2.294608

Pd 13.449510 7.776541 2.334179

Pd 21.523100 7.776542 2.334179

Pd 14.803942 10.103462 2.310241

Pd 22.877531 10.103463 2.310241

Pd 14.798557 5.445513 2.297070

Pd 18.835352 12.437450 2.297070

Pd 16.149195 7.776364 2.325246

Pd 17.486977 10.101517 2.327051

Pd 17.488312 5.439194 2.295195

Pd 21.525107 12.431130 2.295195

Pd 18.837171 7.773547 2.318975

Pd 20.186215 10.096067 2.343794

Pd 20.185733 8.537391 4.726230

Pd 21.562227 10.897619 4.649117

Pd 18.840324 6.212923 4.580329

Pd 22.877120 13.204860 4.580329

Pd 14.800712 8.552035 4.619706

Pd 22.874302 8.552036 4.619706

Pd 16.145299 10.894657 4.626615

Pd 24.218888 10.894659 4.626615

Pd 13.460604 6.220634 4.616947

Pd 17.497399 13.212570 4.616947

Pd 21.534193 6.220635 4.616947

Pd 25.570989 13.212571 4.616947

Pd 17.476777 8.553720 4.667777

Pd 18.817653 10.906448 4.635504

Pd 16.142561 6.219642 4.607392

Pd 20.179356 13.211578 4.607392

C 18.725727 8.308168 7.357103

O 17.608634 8.501294 6.779830

O 19.882169 8.177310 6.859234

H 18.669235 8.247030 8.462624

H 20.191394 10.388443 5.552682

| Bond Pair | Distance (Å) | Distance (Å) from Ref. [81] |
|---|---|---|
| Pd1-H1 | 1.719 | 1.748 |
| Pd2-H1 | 2.027 | 1.878 |
| Pd3-H1 | 1.731 | 1.765 |
| Pd2-O1 | 2.184 | 2.195 |
| Pd4-O2 | 2.117 | 2.133 |
| C-O1 | 1.266 | 1.266 |
| C-O2 | 1.272 | 1.272 |
| C-H2 | 1.109 | 1.111 |

TABLE S1: Bond distances among Pd atoms and those from HCOO, and H.

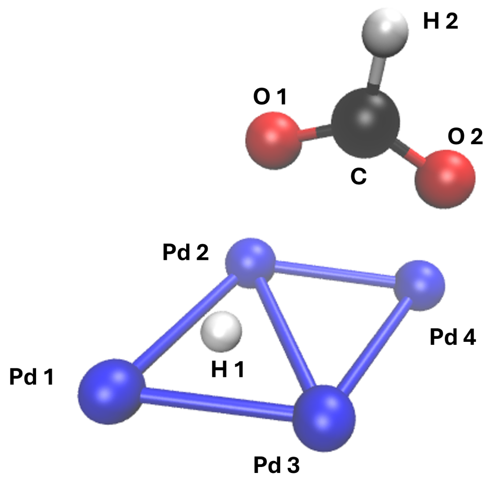

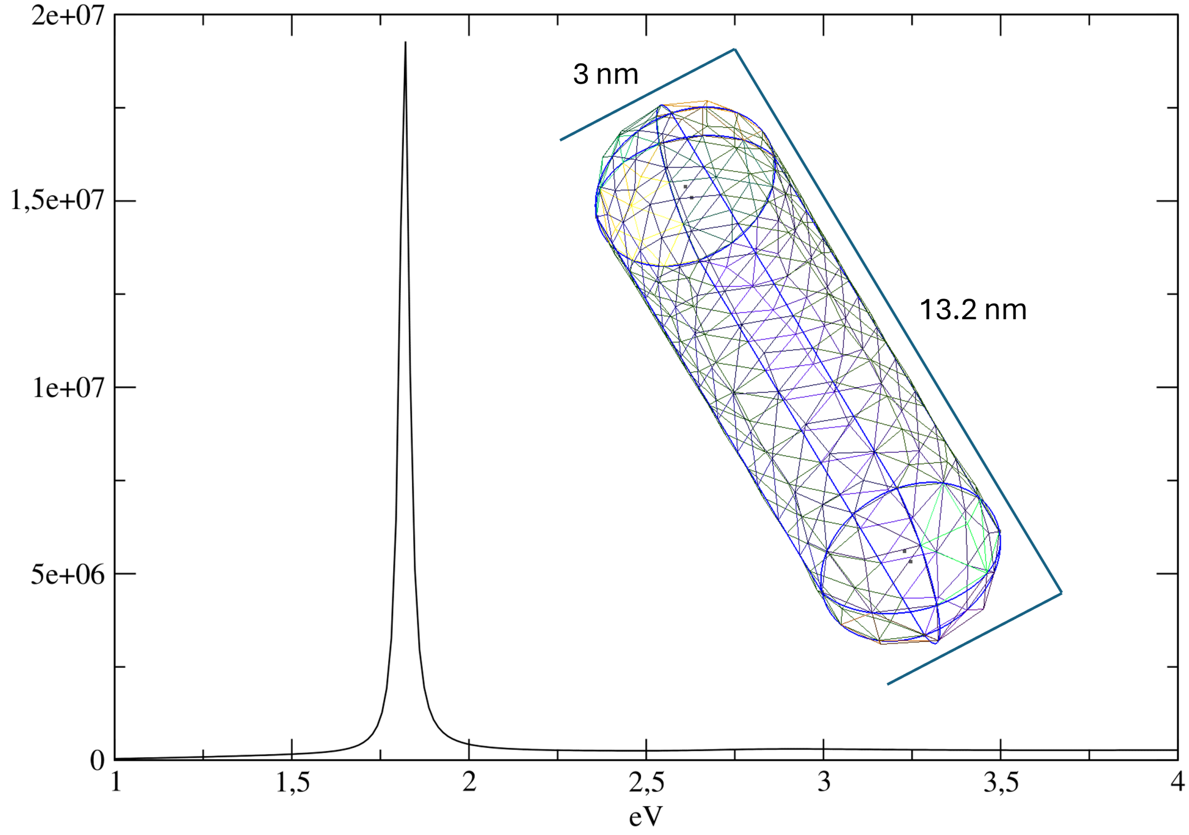


FIG. S1: Absorption spectrum of the Au nanorod of 13 nm full length and 3 nm diameter. An inhomogeneous mesh size was used in the BEM calculation with a total of 504 triangular tesserae. A denser mesh wash used on one of the caps of the nanorod. Gold dielectric function fitted with a sum of Drude-Lorentz terms was used, taken from Ref. 1.

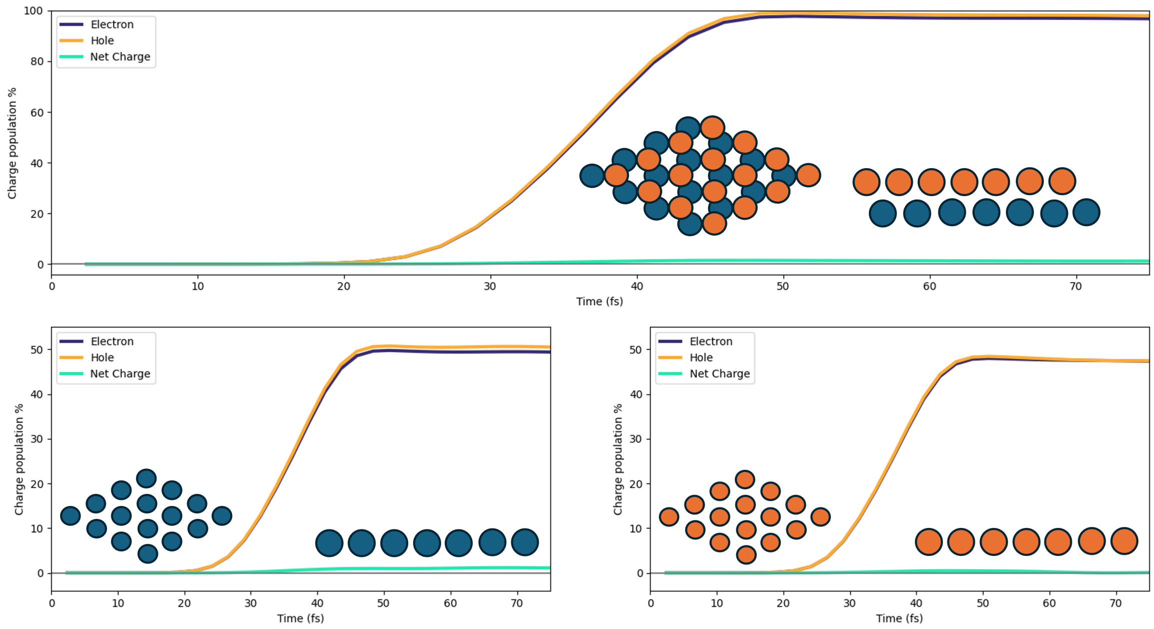


FIG. S2: Upper panel: time evolution of the photoinduced charge populations (electron, hole and net) in the 2L4 system with P1 pulse for the Pd cluster. Bottom panel: time evolution of the photoinduced charge populations (electron, hole and net) of the two lower layers (left) and of the upper layer (right) of Pd atoms.

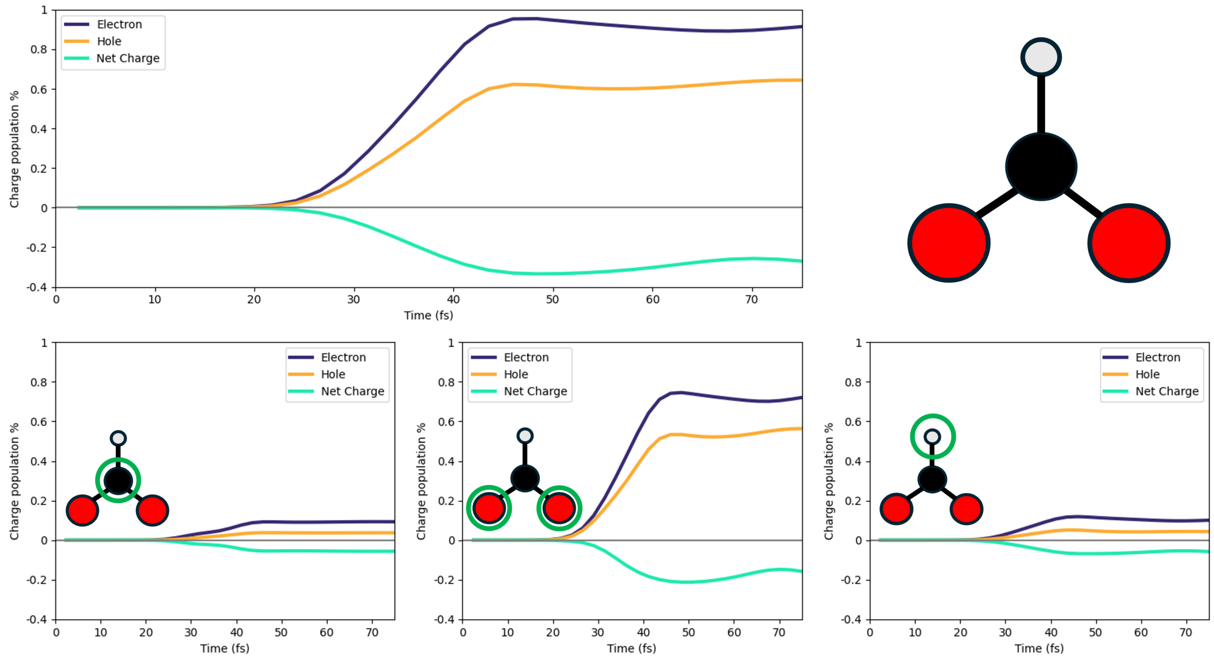


FIG. S3: Upper panel: time-evolution of the photoinduced charge populations (electron, hole and net) of the HCOO moiety in the 2L4 system with P1 pulse. Bottom panel: on the left, time-evolution of the photoinduced charge populations (electron, hole and net) of the carbon atom; in the middle, the same for the oxygen atoms; on the right, the same for the hydrogen atom of HCOO.

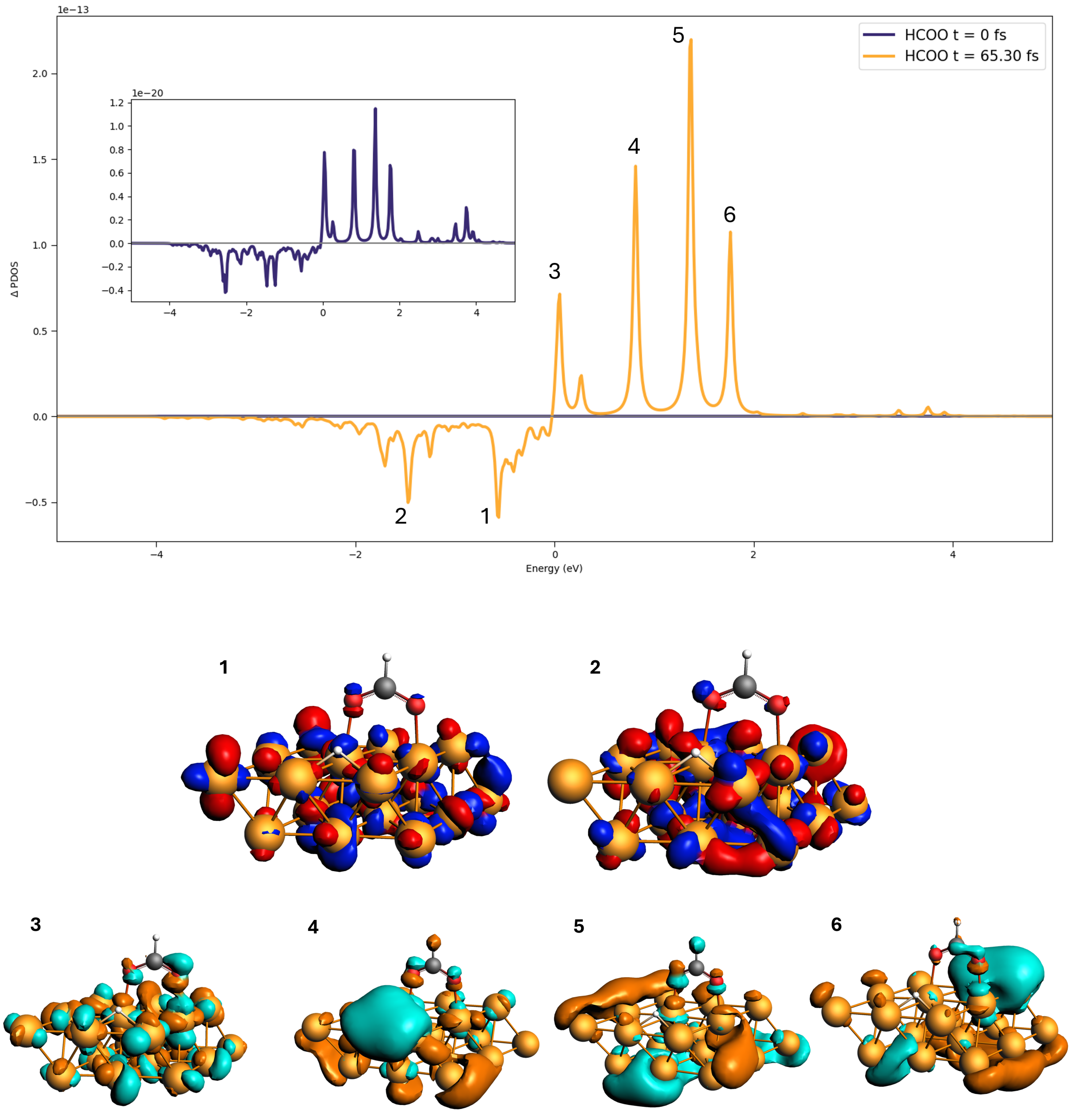


FIG. S4: ΔPDOS of HCOO before (blue line) and after the pulse (yellow line) for the 2L3 system with the P1 pulse. Occupied and virtual orbitals involved in the dynamics are also shown.

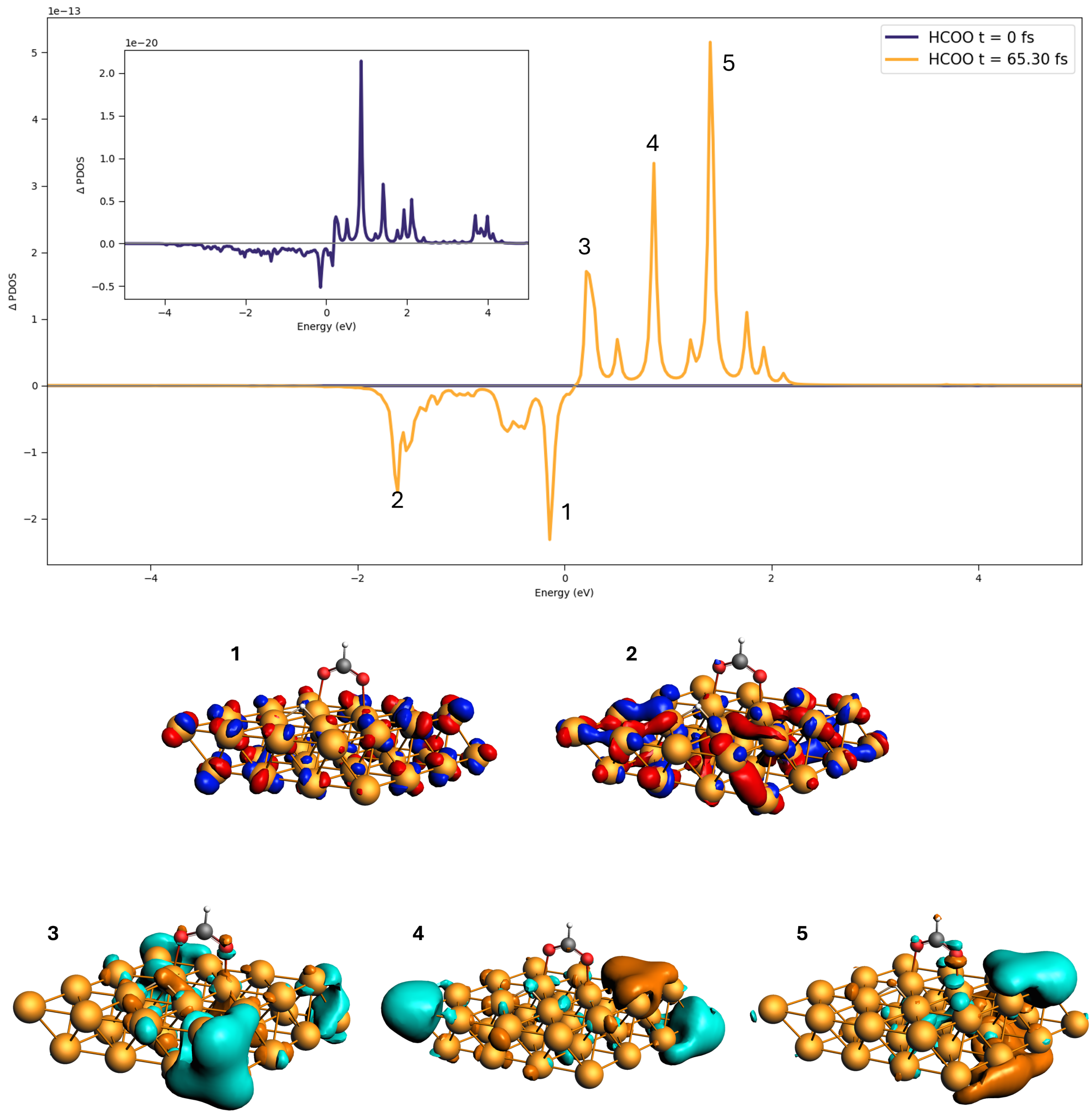


FIG. S5: ΔPDOS of HCOO before (blue line) and after the pulse (yellow line) for the 2L4 system with the P1 pulse. Occupied and virtual orbitals involved in the dynamics are also shown.

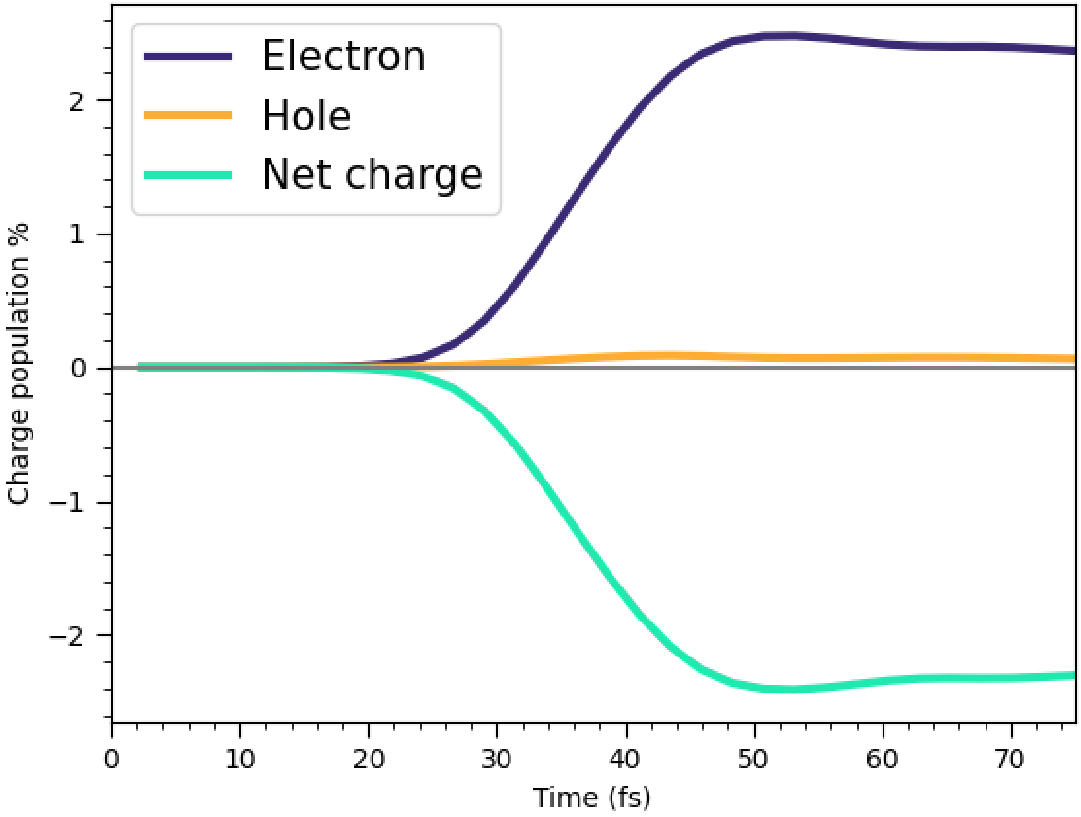


FIG. S6: Time-evolution of the photoinduced charge populations (electron, hole and net) of the H atom adsorbed on the Pd surface in the 2L3 system with P1 pulse.

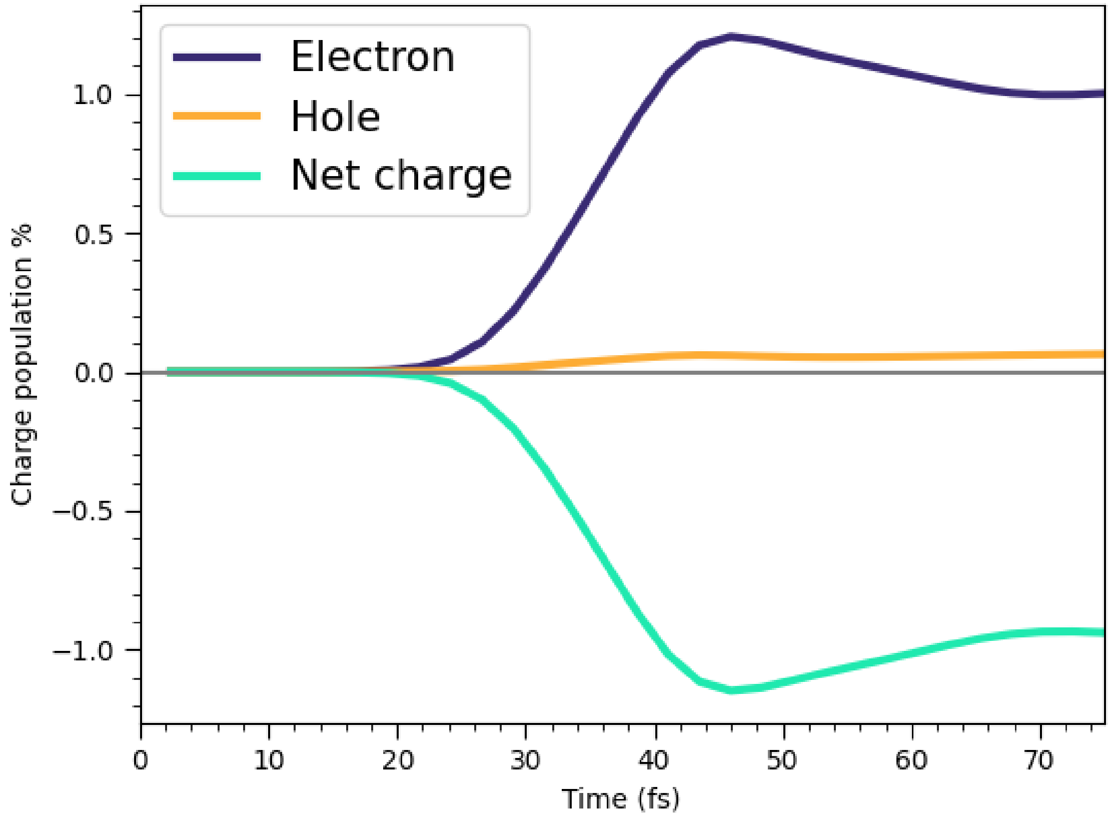


FIG. S7: Time-evolution of the photoinduced charge populations (electron, hole and net) of the H atom adsorbed on the Pd surface in the 2L4 system with P1 pulse.

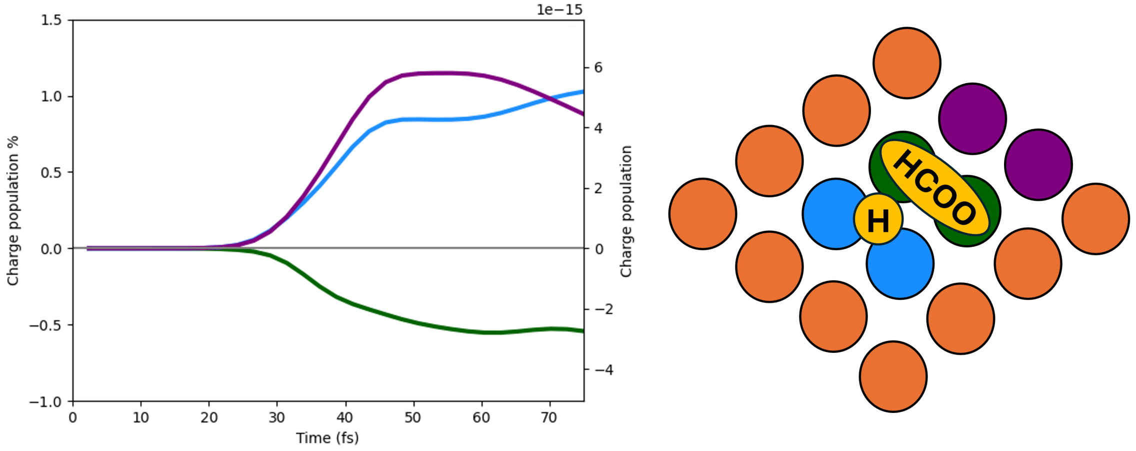


FIG. S8: Time-evolution of the photoinduced net charge population of the Pd atoms of the upper layer in the 2L4 system with P1 pulse. Green: Pd pairs interacting with HCOO. Blue: Pd pairs interacting with H. Purple: Pd pairs not interacting with any molecular species.

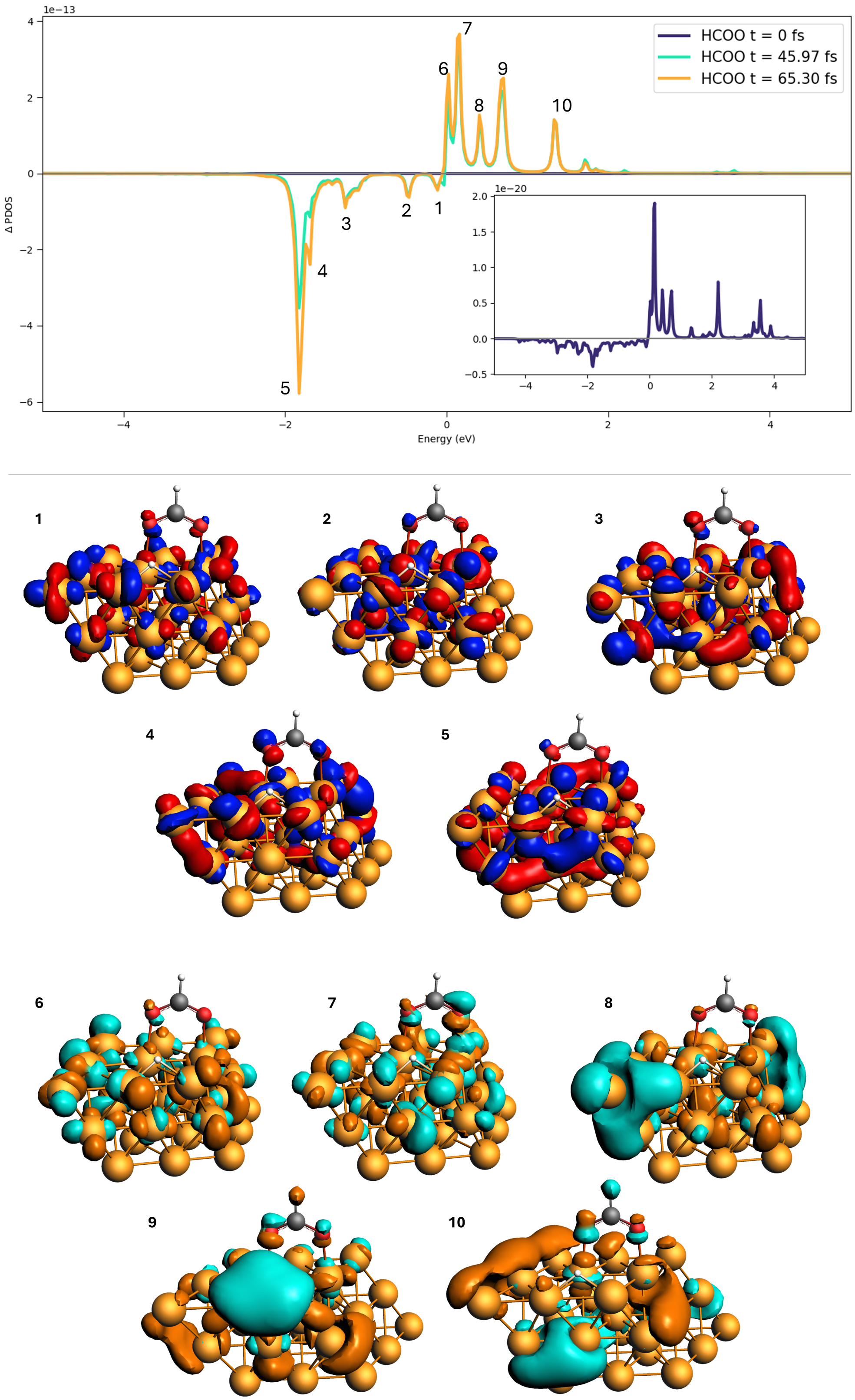


FIG. S9: ΔPDOS of the formate fragment before (blue line) and after the pulse (yellow line) for the 3L3 system with the P1 pulse. The involved occupied and virtual orbitals are also shown.

| Orbitals | $\Delta$PDOS(t=65.50 fs)/$\Delta$PDOS(t=45.97 fs) |
|---|---|
| 1 | 1.21 |
| 2 | 1.11 |
| 3 | 1.03 |
| 4 | 2.09 |
| 5 | 1.63 |
| 6 | 1.37 |
| 7 | 1.12 |
| 8 | 1.16 |
| 9 | 1.16 |
| 10 | 1.00 |

TABLE S2: Ratio between $\Delta$PDOS at 65.50 fs and at 45.97 fs for 2L3 molecular orbitals.

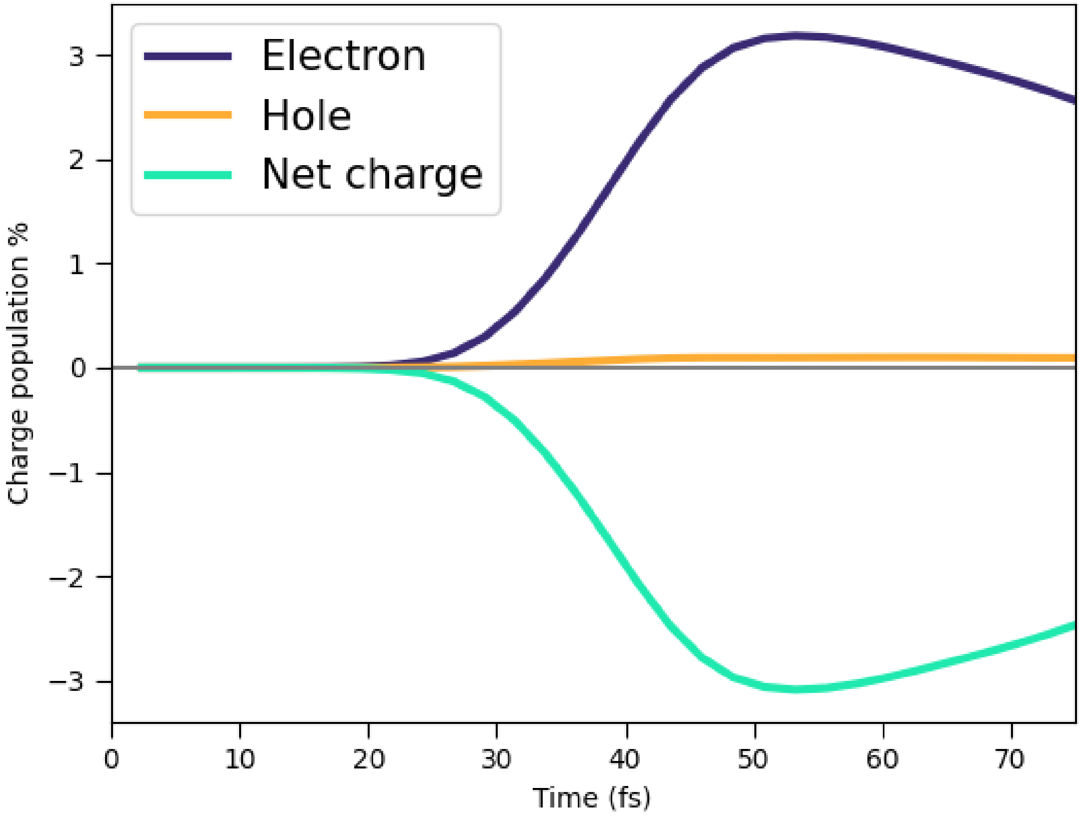


FIG. S10: Time-evolution of the photoinduced charge populations (electron, hole and net) of the H atom adsorbed on the Pd surface in the 3L3 system with P1 pulse.

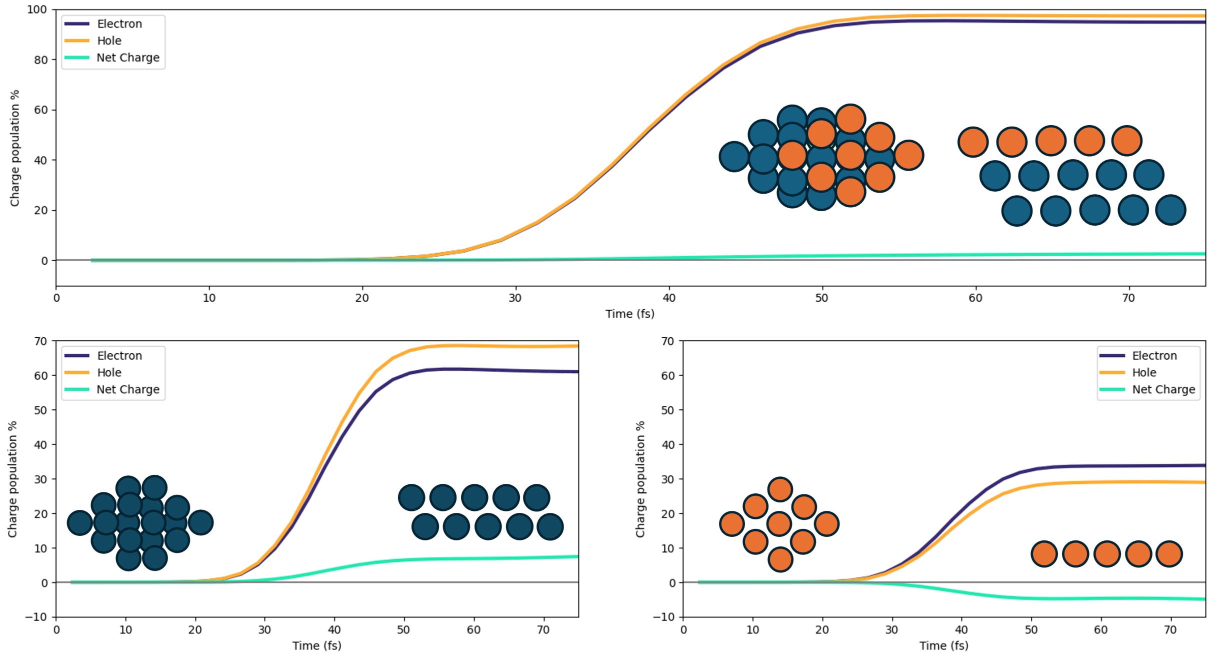


FIG. S11: Upper panel: time-evolution of the photoinduced charge populations (electron, hole and net) of the Pd cluster in the 3L3 system with P2 pulse. Bottom panel: time-evolution of the photoinduced charge populations (electron, hole and net) of the lower layers (left) and of the upper layer (right).

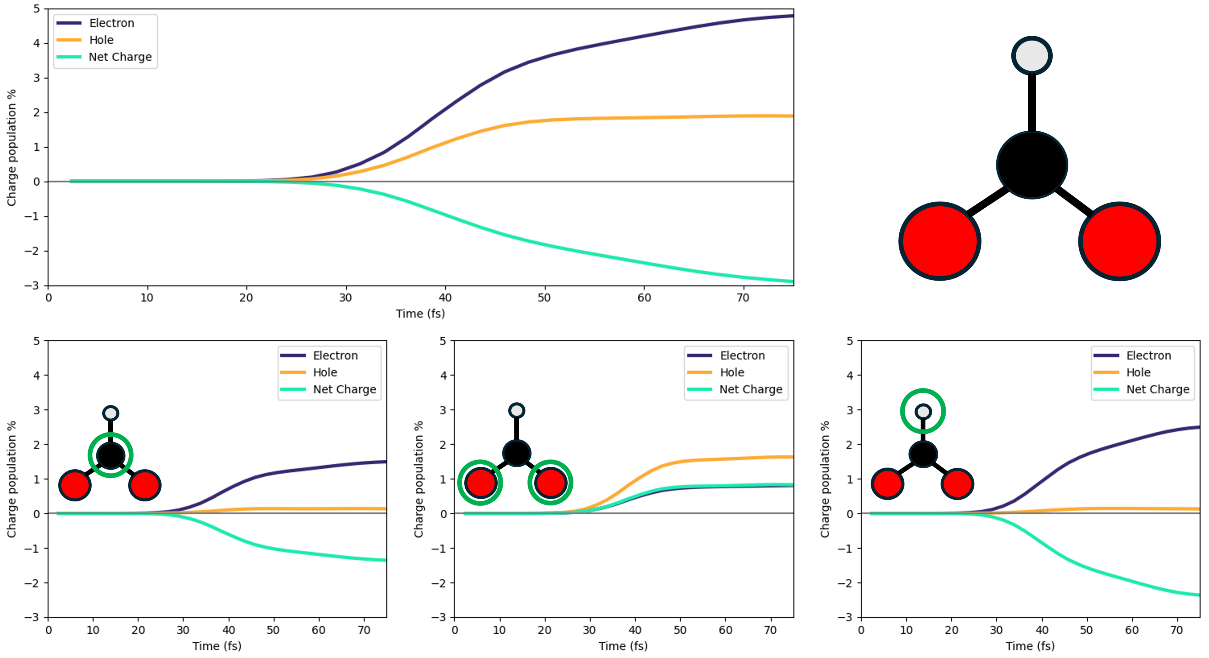


FIG. S12: Upper panel: time-evolution of the photoinduced charge populations (electron, hole and net) of the HCOO fragment in the 3L3 system with P2 pulse. Bottom panel: on the left, time-evolution of the photoinduced charge populations (electron, hole and net) of the carbon atom; in the middle, the same for the oxygen atoms; on the right, the same for the hydrogen atom of HCOO.

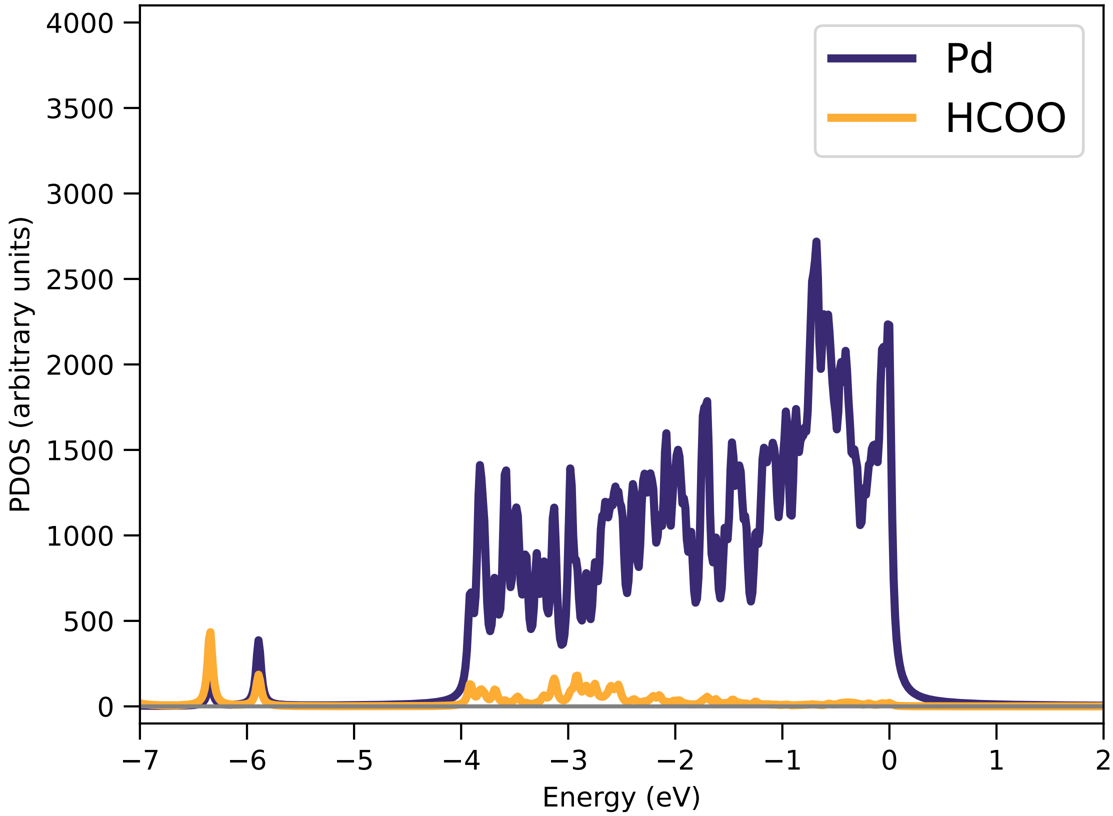


FIG. S13: PDOS of the 2L3 system. PDOS of the Pd atoms is in blue, the PDOS of the HCOO is in yellow.

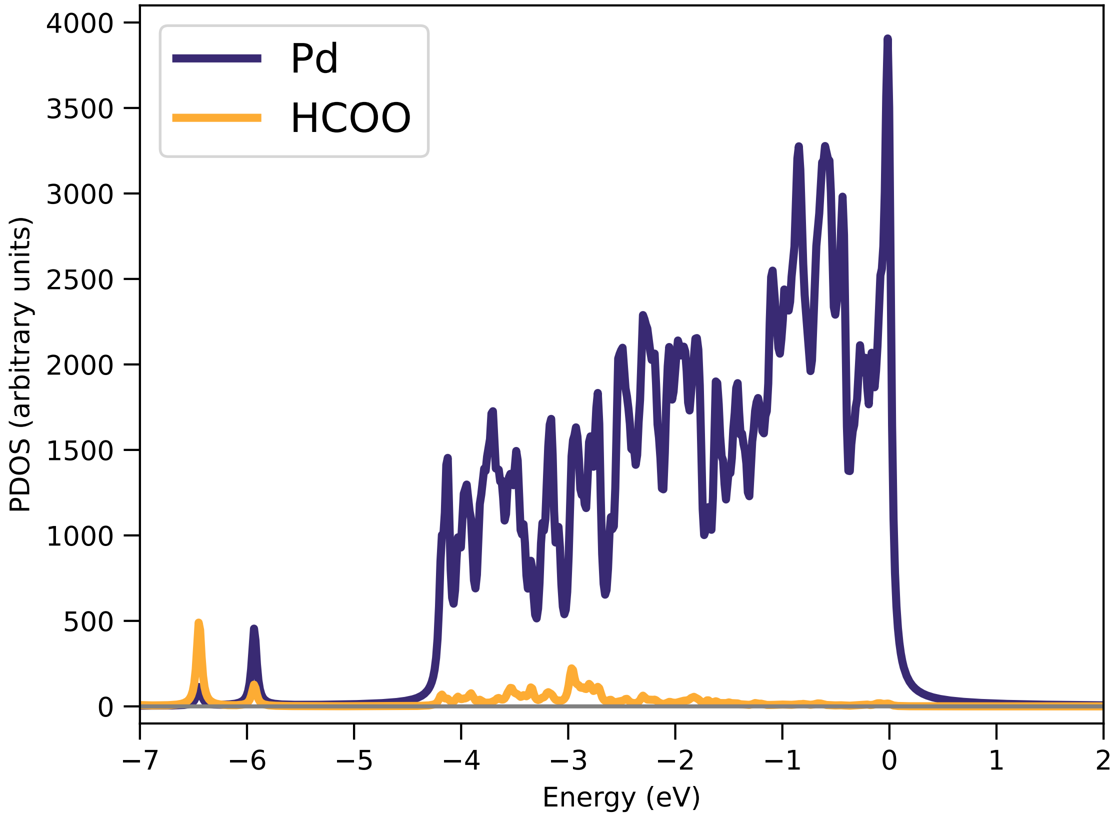


FIG. S14: PDOS of the 3L3 system. PDOS of the Pd atoms is in blue, the PDOS of the HCOO is in yellow.

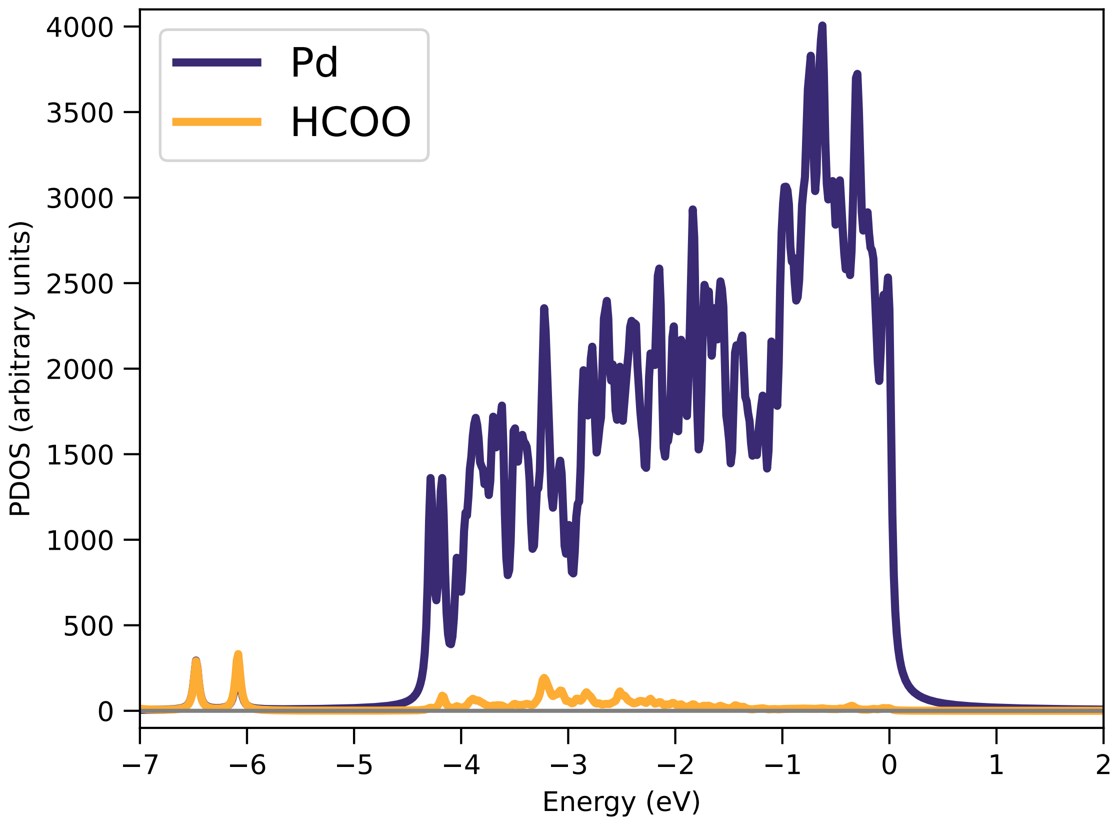


FIG. S15: PDOS of the 2L4 system. PDOS of the Pd atoms is in blue, the PDOS of the HCOO is in yellow.

| | 2L3 | 3L3 | 2L4 |
|---|---|---|---|
| C | 0.361 | 0.357 | 0.357 |
| O | -0.563 | -0.556 | -0.558 |
| O | -0.545 | -0.545 | -0.541 |
| H | 0.270 | 0.289 | 0.272 |
| Tot | -0.477 | -0.455 | -0.470 |
| $H_{ads}$ | 0.031 | 0.013 | -0.033 |

TABLE S3: Ground-state Mulliken charges for HCOO and H in 2L3, 3L3 and 2L4.

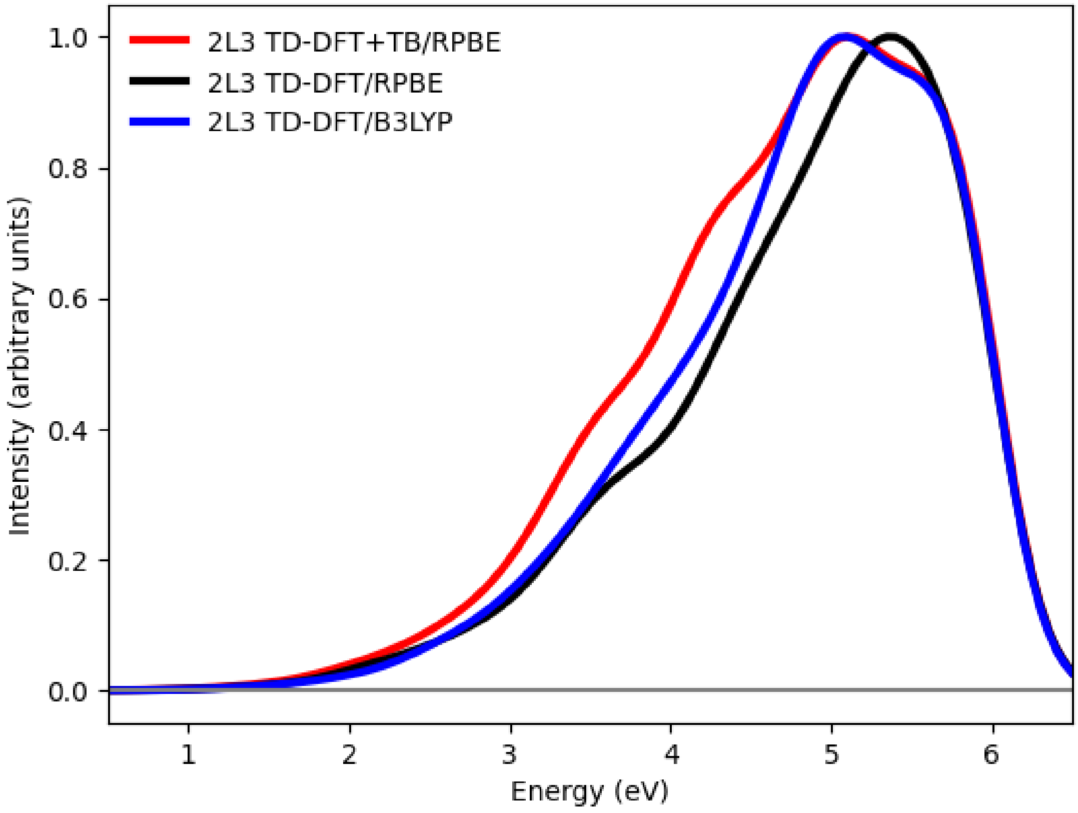


FIG. S16: Absorption spectrum for 2L3 at TD-DFT+TB/RPBE, TDDFT/RPBE and TDDFT/B3LYP level of theory.

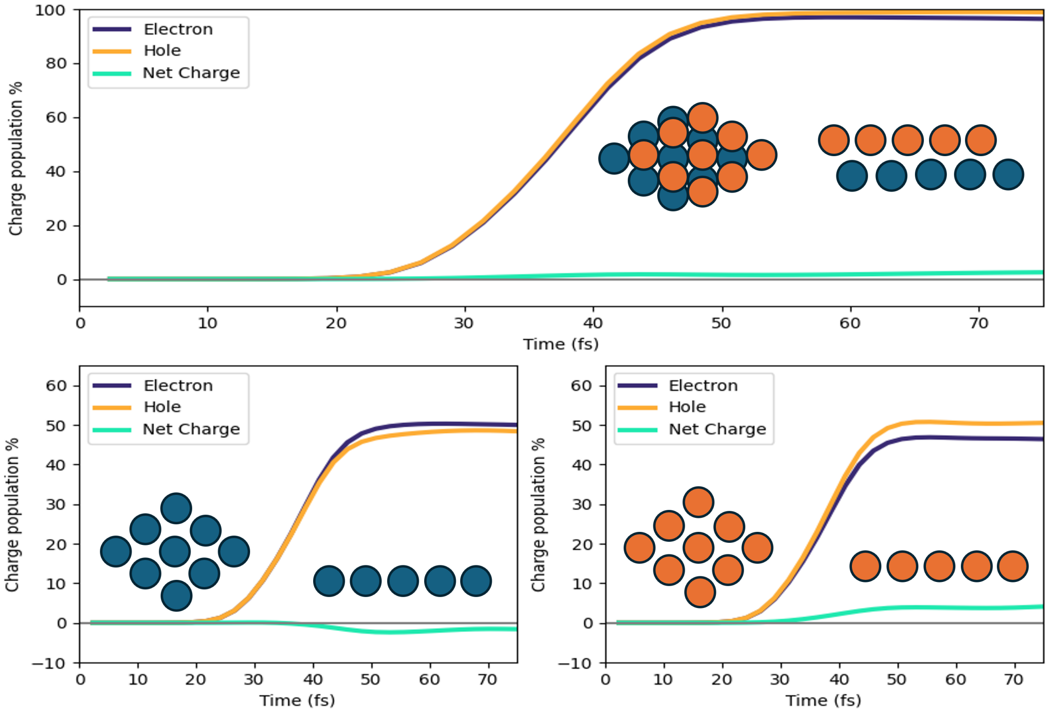


FIG. S17: Time-dependent charge population of the Pd layers for the 2L3 system, using TD-DDFT/B3LYP.

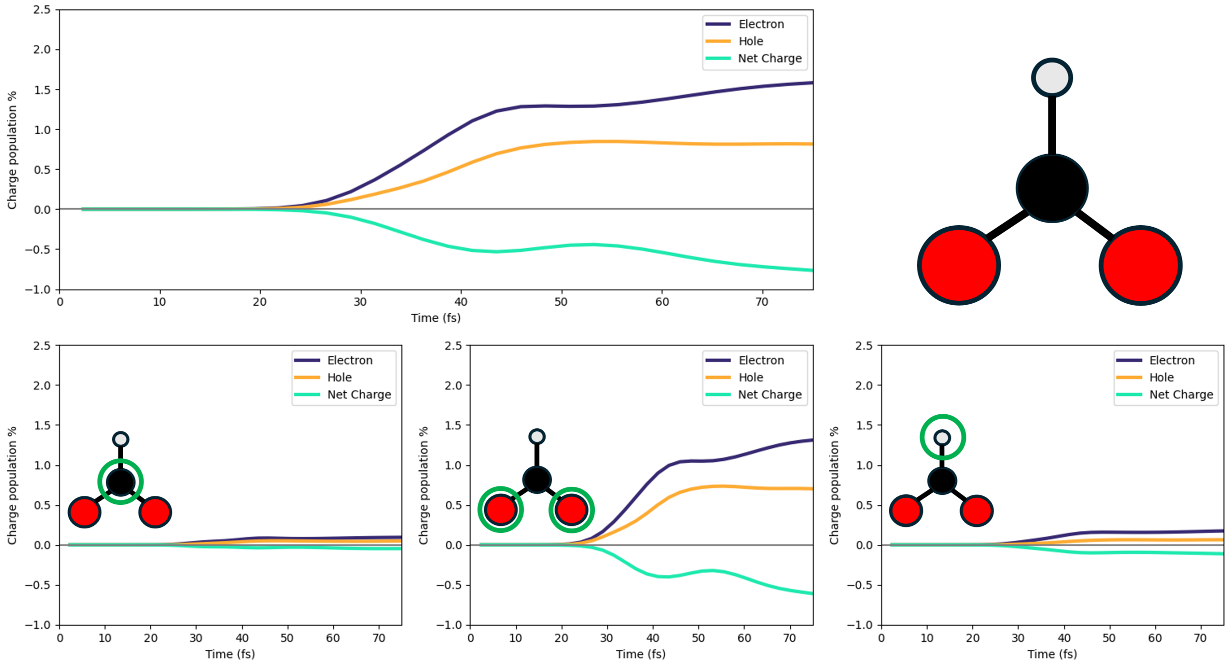


FIG. S18: Time-dependent charge population of HCOO for the 2L3 system, using TD-DDFT/B3LYP.

## REFERENCES

[1]G. Dall'Osto, G. Gil, S. Pipolo, and S. Corni, J. Chem. Phys. **153**, 184114 (2020).
